# Muc2-dependent microbial colonization of the jejunal mucus layer is diet sensitive and confers local resistance to enteric pathogen infection

**DOI:** 10.1016/j.celrep.2023.112084

**Published:** 2023-02-06

**Authors:** George M.H. Birchenough, Bjoern O. Schroeder, Sinan Sharba, Liisa Arike, Christian V. Recktenwald, Fabiola Puértolas-Balint, Mahadevan V. Subramani, Karl T. Hansson, Bahtiyar Yilmaz, Sara K. Lindén, Fredrik Bäckhed, Gunnar C. Hansson

**Affiliations:** 1 Department of Medical Biochemistry & Cell Biology, Institute of Biomedicine, University of Gothenburg, Gothenburg, Sweden; 2 Wallenberg Centre for Molecular & Translational Medicine, University of Gothenburg, Gothenburg, Sweden; 3 Department of Molecular Biology and Laboratory for Molecular Infection Medicine Sweden (MIMS), Umeå University, Umeå, Sweden; 4 Wallenberg Laboratory, Department of Molecular and Clinical Medicine, Institute of Medicine, University of Gothenburg, Gothenburg, Sweden; 5 Department for BioMedical Research, University of Bern, Bern, Switzerland; 6 These authors contributed equally; 7 Lead contact

## Abstract

Intestinal mucus barriers normally prevent microbial infections but are sensitive to diet-dependent changes in the luminal environment. Here we demonstrate that mice fed a Western-style diet (WSD) suffer regiospecific failure of the mucus barrier in the small intestinal jejunum caused by diet-induced mucus aggregation. Mucus barrier disruption due to either WSD exposure or chromosomal *Muc2* deletion results in collapse of the commensal jejunal microbiota, which in turn sensitizes mice to atypical jejunal colonization by the enteric pathogen *Citrobacter rodentium*. We illustrate the jejunal mucus layer as a microbial habitat, and link the regiospecific mucus dependency of the microbiota to distinctive properties of the jejunal niche. Together, our data demonstrate a symbiotic mucus-microbiota relationship that normally prevents jejunal pathogen colonization, but is highly sensitive to disruption by exposure to a WSD.

## In brief

Birchenough et al. characterize regiospecific small intestinal mucus layer disruption after exposure to a Western-style diet. They show dependency of the jejunal microbiota on the presence of an intact mucus layer and demonstrate that mucus supports resistance against pathogen colonization by providing a habitat for the microbiota.

## Graphical abstract

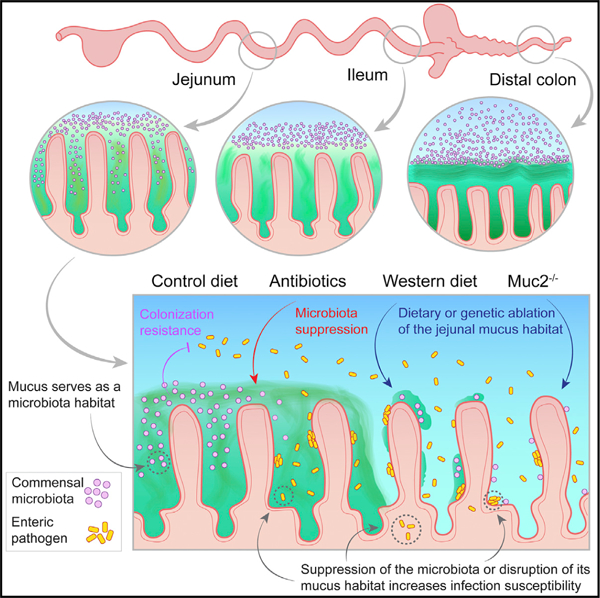

## INTRODUCTION

Exposure to a Western-style diet (WSD) is a key driver of the obesity pandemic. Originally confined to developed countries, increasing levels of obesity have been observed in developing regions with higher concomitant infectious disease burdens.^1^ Evidence suggests that obese and diabetic humans are at higher risk of infection, especially at mucosal surfaces.^2–4^ It is thus of importance to understand how the Western-style diet modulates the functions of our mucosal defensive systems.

Mucus secreted by epithelial goblet cells is a critical element of mucosal defense against infection, particularly in the gastrointestinal tract. Goblet cells secrete large polymers of the gel-forming mucin Muc2, which are the structural backbone of the mucus layers that coat the epithelial surface.^5^ These form barrier systems that vary at different locations along the gastrointestinal (GI) tract.^6,7^ In the distal colon, densely packed Muc2 polymers generate an inner mucus layer structure that physically restricts microbial access to the epithelium.^8^ In the small intestine a looser mucus layer concentrates antimicrobial proteins secreted from epithelial enterocytes and Paneth cells, creating a bactericidal gradient that also prohibits epithelial contact with live microbes.^6,9^

Colonic mucus barrier function is impacted by factors associated with diet^10–12^ and host metabolism.^13,14^ In mice, WSD-induced deterioration of mucus has been causally linked to the microbiota due to a lack of complex dietary polysaccharides.^11,12^ Furthermore, loss of mucus barrier function results in hyper-susceptibility to infection, inflammation, and tumor formation.^15–17^ The links among diet, the microbiota, and the mucus barrier in the colon and their potential impact on health are thus established. However, little attention has been focused on the small intestinal mucus barrier, despite this region being a common site of infection and the primary interaction site for the diet with the mucosa.

## RESULTS

### Exposure to a WSD drives regiospecific small intestinal mucus barrier dysfunction

**Figure 1.**
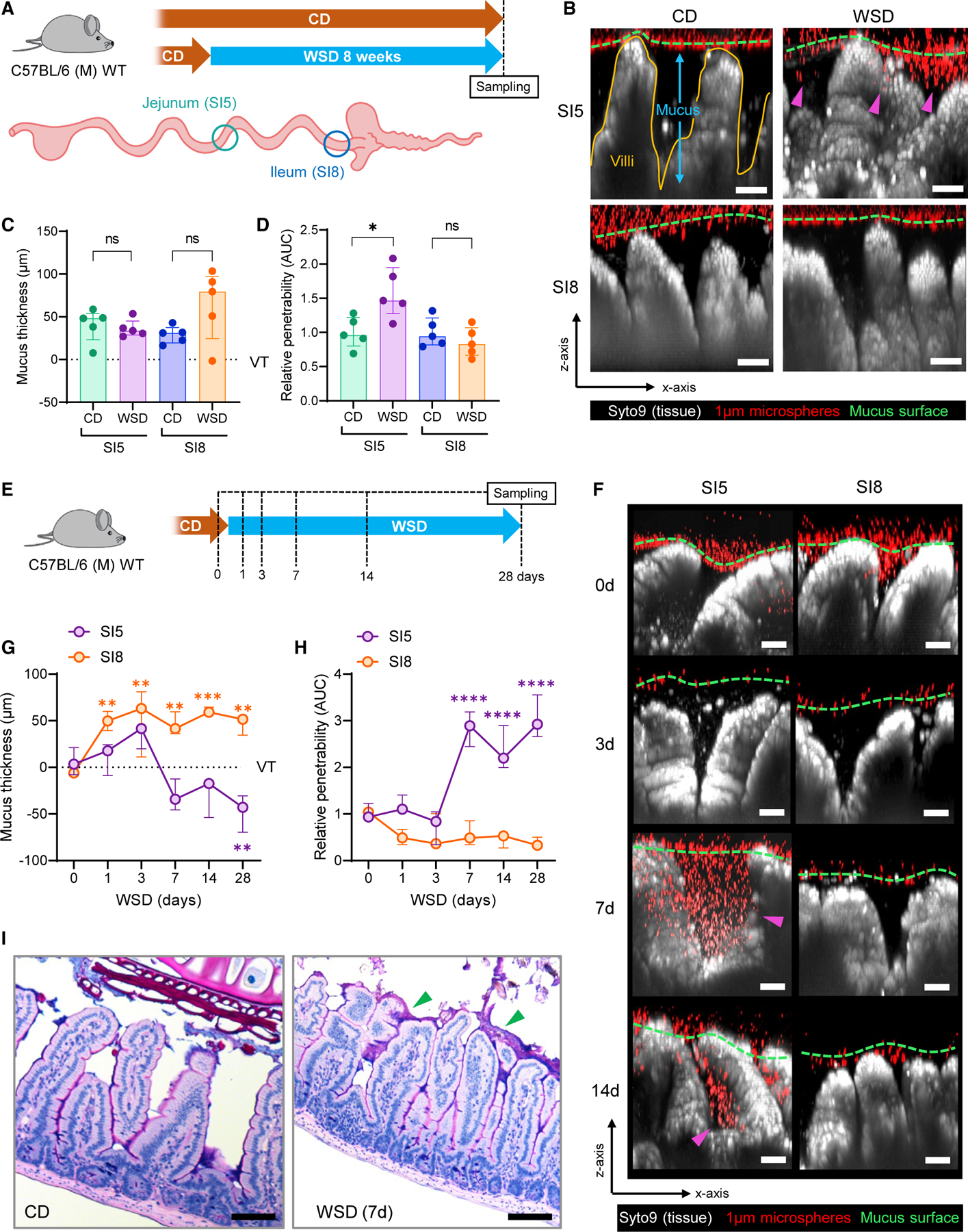
WSD induces jejunal mucus barrier dysfunction (A) Schematic illustrating CD and WSD experimental groups and intestinal tissue sampling points. (B) Confocal z stacks showing x/z axis cross sections of *ex vivo* SI5 and SI8 tissue (gray) and 1-µM microspheres (red) from CD and WSD-fed mice; approximate mucus surface (green dashed line) and microspheres penetrating into mucus (magenta arrows) indicated. (C and D) Quantification of mucus thickness (C) relative to villus tips (VTs) and relative barrier function (D) based on data extracted from images shown in (B). Data in (D) are normalized to CD group mean. (E) Schematic illustrating sampling points for WSD time course. (F) Confocal z stacks showing x/z axis cross sections of SI5 and SI8 tissue (gray) and 1-µM microspheres (red) from WSD-fed mice at different time points; approximate mucus surface (green dashed line) and microspheres penetrating into mucus (magenta arrows) indicated. (G and H) Quantification of mucus thickness (G) relative to villus tips (VTs) and relative barrier function (H) based on data extracted from images shown in (F). Data in (H) are normalized to day 0 group mean. (I) Alcian blue/periodic acid-Schiff (AB/PAS)-stained fixed tissue sections from CD and WSD-fed mice. Images are representative of n = 5/group. All image scale bars are 50 µM. Data show median and interquartile range for n = 5 (C, D) and n = 3 (G, H) mice per group. Significance by Mann-Whitney (C, D) or Dunnett’s (G, H) test (*p < 0.05, **p < 0.01, ***p < 0.001, p < 0.0001, ns: not significant).

To define the impact of diet on small intestinal mucus barrier properties, we fed mice a WSD and compared them with animals fed a normal chow diet (CD). Mice from both CD and WSD groups were killed after 8 weeks. Tissues were obtained from the mid-jejunal (SI5) and terminal ileal (SI8) regions (Figure 1A) and mucus properties were analyzed using a microsphere penetration assay for quantification of mucus thickness and barrier function.^18^ We observed no significant effect of WSD exposure on mucus thickness in either SI5 or SI8 (Figures 1B and 1C). Conversely, a significant increase in SI5, but not SI8, mucus penetrability was detected in WSD compared with CD-fed mice (Figures 1B and 1D).

We assessed the kinetics of SI5 mucus barrier dysfunction (Figure 1E) and observed a decrease in SI5 mucus thickness and increase in mucus penetrability that occurred between 3 and 7 d after WSD exposure (Figures 1F–1H). WSD-induced SI5 mucus barrier dysfunction was maintained until 28 d WSD, and was characterized by microspheres penetrating through the mucus to the base of the villi (Figure 1F).

Assessment of small intestinal mucus properties in fixed tissue sections is challenging due to the variable preservation of mucus in fixed tissue sections. Nevertheless, we examined Alcian blue/periodic acid-Schiff (AB/PAS)-stained fixed tissue sections from CD and WSD-fed mice to determine if WSD exposure resulted in alterations in fixed SI5 tissues or mucus (Figure 1I). As expected in SI5, mucus preservation was poor in CD-fed mice; however, in WSD-fed mice we identified a discontinuous layer of intensely stained mucus, indicating that WSD-induced alterations may alter mucus properties and affect preservation.

Consequently, these results demonstrated that WSD exposure over a relatively short period had a deleterious effect on jejunal (SI5) but not ileal (SI8) mucus properties, thus demonstrating a regiospecific effect of diet on mucus barrier function in the small intestine.

### The role of the microbiota in WSD-induced jejunal mucus barrier dysfunction

**Figure 2.**
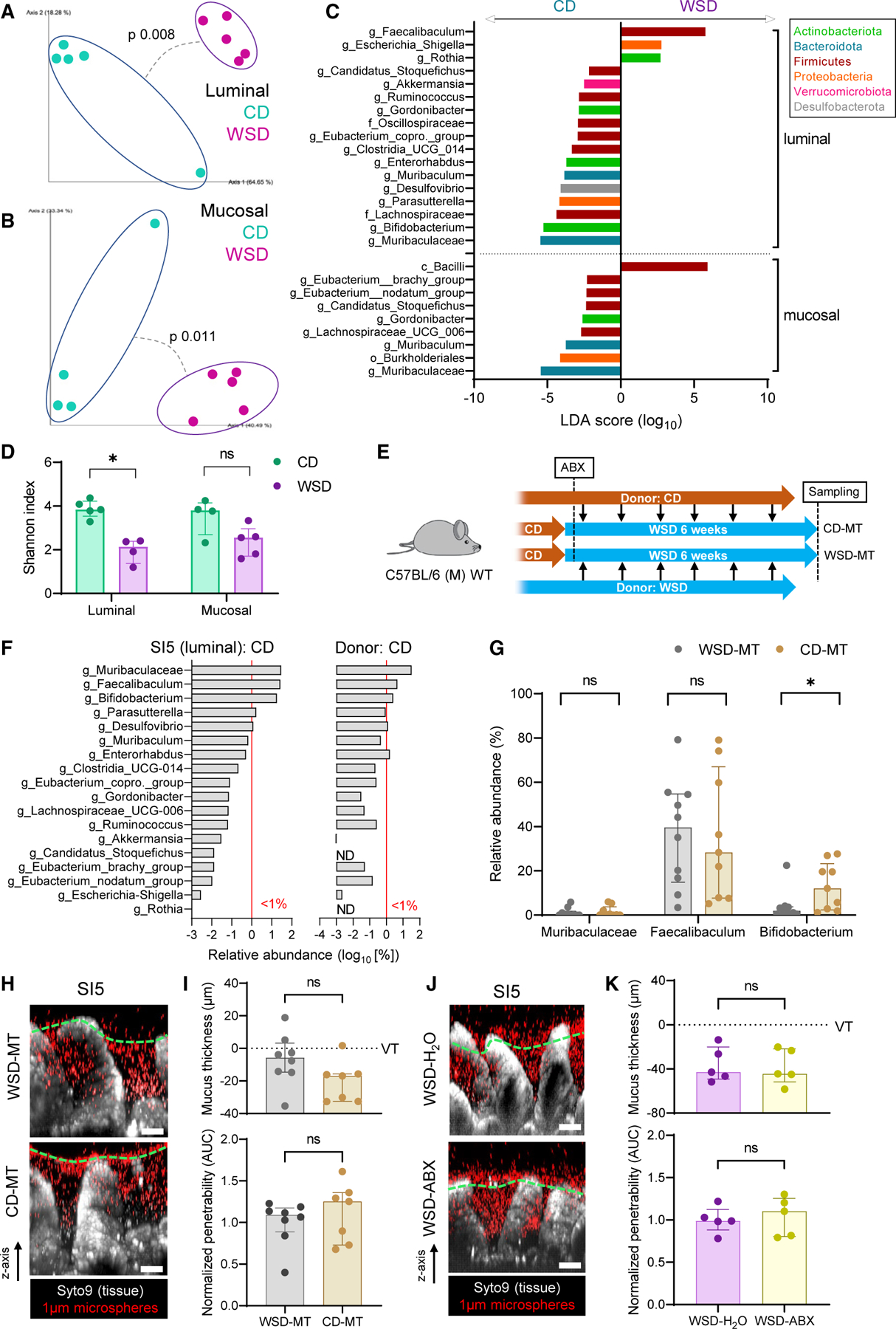
The role of the microbiota in WSD-induced mucus barrier dysfunction (A–D) Analysis of microbiota community structure in SI5 lumen and mucosa of CD and 8-week WSD-fed mice by 16S rRNA gene sequencing. Principal-component analysis of β-diversity (Bray-Curtis dissimilarity) of bacterial communities in the luminal (A) and mucosal (B) compartments. Linear discriminant analysis (LDA) effect size identification of diet-enriched bacterial taxa (C). α-diversity (Shannon index) of bacterial communities (D). (E) Schematic illustrating CD and WSD microbiota transfer (MT) into WSD-fed mice after antibiotics (ABX) treatment. (F) Median relative abundance of all genus-level bacterial taxa impacted by WSD exposure (C) in the CD-fed SI5 microbiota (left panel) and CD-fed donor caecal contents microbiota (right panel) used for CD-MT gavage. Red line indicates a relative abundance of 1%. (G) Relative abundance of three major bacterial taxa found in the SI5 microbiota in WSD-MT and CD-MT SI5 samples. (H) Confocal z stacks showing x/z axis cross sections of *ex vivo* SI5 tissue (gray) and 1-µm microspheres (red) from WSD-MT and CD-MT mice; approximate mucus surface (green dashed line) indicated. (I) Quantification of mucus thickness (upper) relative to villus tips (VTs) and relative barrier function (lower) based on data extracted from images shown in (H). Barrier function data are normalized to the WSD-MT group mean. (J) Confocal z stacks showing x/z axis cross sections of *ex vivo* SI5 tissue (gray) and 1µm microspheres (red) from WSD-fed mice with (ABX) or without (H_2_O) supplemented in their drinking water; approximate mucus surface (green dashed line) indicated. (K) Quantification of mucus thickness (upper) relative to villus tips (VTs) and relative barrier function (lower) based on data extracted from images shown in (J). Barrier function data are normalized to the WSD-H_2_O group mean. All image scale bars are 50 µm. Data show median and interquartile range for n = 4–5 (A–D, J–K) or n = 9–10 (E–I) mice per group. Significance by pairwise PERMANOVA (A, B) or Mann-Whitney (D, G, I, K) test (*p < 0.05, ns: not significant).

Studies have causally linked diet or obesity-induced mucus barrier deterioration in the large intestine (colon) to alterations in the microbiota.^11–13^ We therefore sought to characterize the potential role of the microbiota in SI5. We investigated the composition of the microbiota in the SI5 luminal and mucosal compartments using 16S rRNA gene sequencing. Comparison of 16S beta diversity (Bray-Curtis dissimilarity) indicated significant diet-dependent divergence in microbiota community structure in both the luminal and mucosal compartments (Figures 2A and 2B). To establish which 16S amplicon sequence variants (ASVs) significantly correlated with the different diets, we employed linear discriminant analysis effect size (LEfSe) analysis (Figure 2C). This identified enrichment of some taxa in WSD-fed mice, while most discriminant taxa were depleted compared with CD-fed mice. The trend toward decreased taxon abundance in WSD-fed mice was reflected in a significant decrease in alpha diversity (Figure 2D). Differences between CD and WSD-fed mice were largely driven by substantial shifts in the Muribaculaceae, *Faecalibaculum* and *Bifidobacterium* bacterial genera that comprised the bulk (combined average 74.4%) of ASVs in CD-fed mice, with significant depletion of Muribaculaceae and *Bifidobacterium* and enrichment of *Faecalibaculum* in the WSD-exposed microbiota.

While our data demonstrated that WSD-induced SI5 mucus barrier dysfunction coincided with shifts in local microbiota community structure, the causal role of these alterations remained unclear. We thus performed microbiota transplant experiments (Figure 2E) in which mice were fed WSD for 6 weeks while receiving microbiota transplants from caecal content of CD-fed mice (CD-MT) or WSD-fed mice (WSD-MT). Most bacterial taxa normally detected in the SI5 of CD-fed mice were detected in the caecal donor material, at both genus (Figure 2F) and species level (Figure S1A); however, SI5 microbiota β-diversity in CD-MT and WSD-MT mice failed to identify any divergence in overall community structure (Figure S1B), indicating that CD microbiota transfer was not able to reverse WSD-induced alterations. Of the three major WSD-sensitive bacterial taxa, a significant difference was only observed for *Bifidobacterium*, which was enriched in CD-MT compared with WSD-MT (Figure 2G), indicating that this bacterial taxon was successfully engrafted in CD-MT mice. However, SI5 mucus thickness and penetrability were unaltered in CD-MT compared with WSD-MT, indicating that *Bifidobacterium* was not sufficient to prevent WSD-induced mucus dysfunction (Figures 2H and 2I).

We further tested microbiota causality by treating CD-fed mice with a combination of broad-spectrum antibiotics before being switched to WSD. SI5 mucus properties after 7-d WSD exposure were similar in both vehicle (H_2_O)- and antibiotic (ABX)-treated groups (Figures 2J and 2K), indicating the depletion of the microbiota had no effect on WSD-induced mucus dysfunction.

Consequently, the jejunal microbiota is highly sensitive to dietary alterations; however, unlike colon, the jejunum is refractive to recolonization by CD-associated bacteria, suggesting that WSD-induced alterations in the jejunal environment inhibit this process. Furthermore, the causal link between the microbiota and diet-dependent colonic mucus barrier disruption was not replicated in relation to jejunal mucus barrier dysfunction, suggesting an alternative mechanism for this phenomenon in the small intestine.

### WSD-induced barrier dysfunction results from mucus aggregation

**Figure 3.**
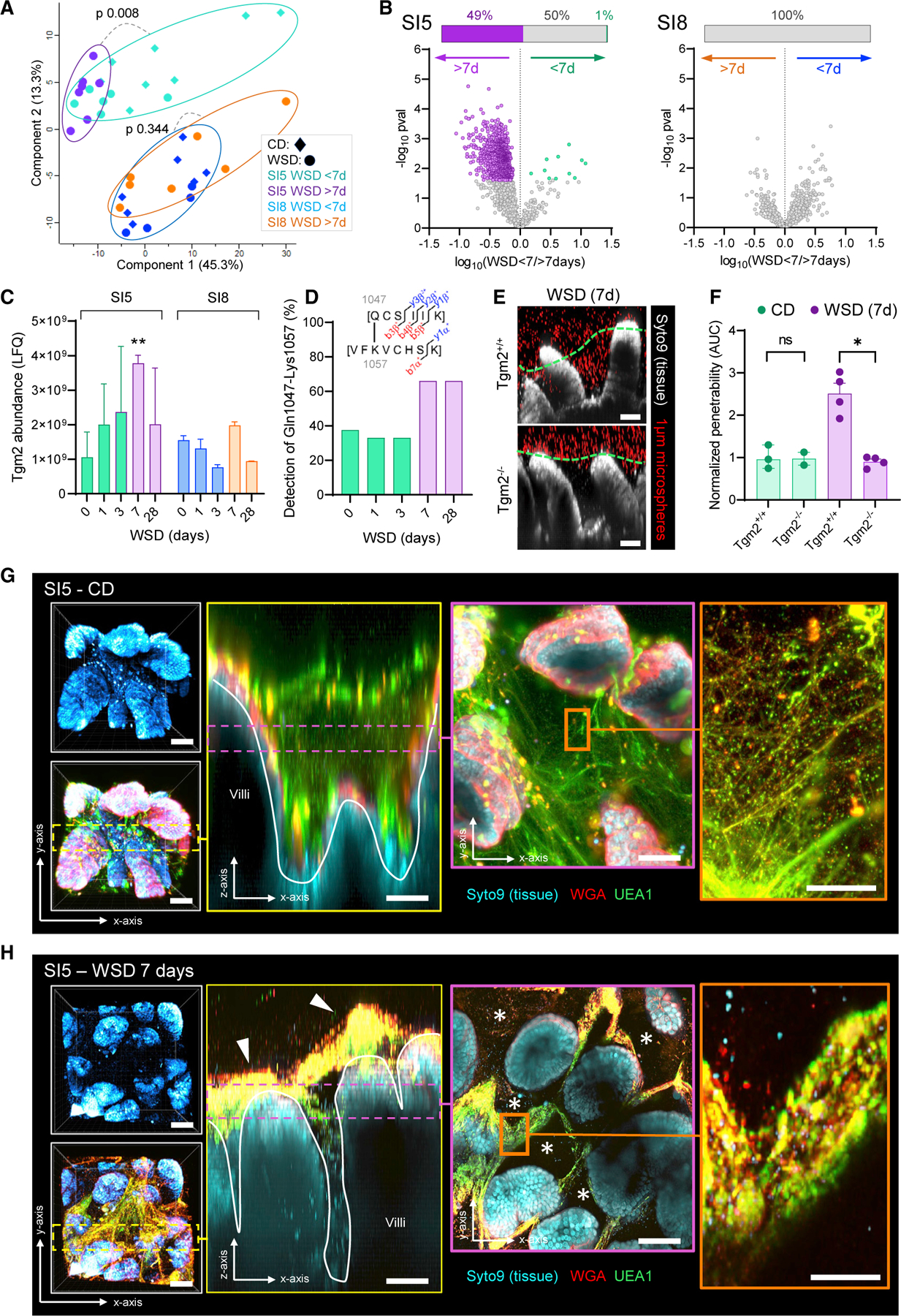
WSD induces jejunal mucus aggregation (A–C) Mass spectrometry-based label-free quantification (LFQ) of SI5 and SI8 mucus proteomes from CD and WSD-fed mice. Principal-component analysis (A) and volcano plots (B) comparing protein abundance from mice fed WSD >7 d or WSD <7 d. Inset bars in B show proportion of significant (colored) or non-significant (gray) differential proteins between groups after correction for multiple testing. Abundance of Tgm2 in SI5 and SI8 mucus at different times after WSD exposure (C). (D) Proportion of SI5 mucus samples from WSD-fed mice at different time points where the Gln-1047-Lys1057 cross-linked peptide (illustrated in figure inset) was detected. (E) Confocal z stacks showing x/z axis cross sections of *ex vivo* SI5 tissue (gray) and 1-µm microspheres (red) from WSD-fed *Tgm2*^+/+^ and *Tgm2*^−/−^ mice; approximate mucus surface (green dashed line) indicated. (F) Quantification of relative SI5 mucus barrier function based on data extracted from images shown in (E). Barrier function data is normalized to the *Tgm2*^+/+^ CD-fed group mean. (G) *Ex vivo* confocal microscopy imaging of SI5 tissues (blue) and mucus structure using fluorophore conjugated UEA1 (green) and WGA (red) lectins in CD-fed mice. Confocal z stacks showing x/y axis projections (gray panels) and an x/z axis cross section (yellow panel; yellow dashed line). Confocal z stacks showing low magnification (purple panel; purple dashed line) and high magnification (orange panel) x/y axis cross sections. (H) *Ex vivo* confocal microscopy imaging of SI5 tissues and mucus structure in mice fed WSD for 7 d using the same approach described for (G). Images show x/y axis projections (gray box), x/z axis cross section (yellow panel; yellow dashed line) and x/y axis cross section (purple panel; purple dashed line) and high magnification (orange panel) x/y axis cross sections. Mucus aggregates (white arrows in yellow panel) and gaps in the mucus structure (asterisks in purple panel) are indicated. All image scale bars are 50 µm, with the exception of the orange panels in G/H (10 µm). Data show median and interquartile range for n = 2–8 (C) or n = 2–4 (F) mice per group as indicated. Significance by pairwise PERMANOVA (A), Welch’s t test and Permutation-based false discovery rate (B, C), Dunnett’s test (C) or Mann-Whitney (*p < 0.05, **p < 0.01).

To investigate the cause(s) of WSD-induced SI5 mucus barrier dysfunction, we employed mass spectrometry-based proteomic analysis of SI5 and SI8 mucus samples collected from CD and WSD-fed mice. Principal-component analysis of proteomic data indicated that samples clustered based on small intestinal region rather than diet (Figure 3A). In order to establish if alterations in the SI5 mucus proteome corresponded with mucus dysfunction, we compared mice fed WSD for >7 d (penetrable mucus) to mice fed WSD for <7 d (impenetrable mucus) (Figure 3B). Comparison of SI5 from these groups resulted in highly skewed data, with a large number (49% proteins) enriched in the penetrable compared with impenetrable mucus group (Figure 3B; Table S1). This enrichment was specific to SI5 and was not observed in an identical comparison of SI8 samples (Figure 3B; Table S2) or from SI5 mucus obtained from microbiota transfer experiments (Figures S2A and S2B). Enriched SI5 proteins were not distinguished by overall abundance or molecular mass (Figures S2C and S2D). Notably, proteins altered in penetrable SI5 mucus did not include most of the highly abundant core mucus components with established or suggested roles in maintaining mucus function (Figure S2E). Consequently, our data indicated that WSD-induced SI5 mucus barrier dysfunction was not clearly linked to alterations in the core mucus proteome, but coincided with enrichment of a large fraction of SI5 mucus proteins.

Mucus properties are regulated by numerous factors that remain poorly understood; however, several enzymes have been implicated in Muc2 expansion (Mep1a, Mep1b), processing (Clca1), and isopeptide cross-linking (Tgm2, Tgm3).^19–21^ In order to determine if enzymatic alterations correlated with mucus properties, we determined their abundance at different time points post-WSD feeding. No differences in Mep1a or Mep1b were detected, while a transient increase in Clca1 was identified in SI8, but not SI5, after 1-d WSD exposure (Figures S2F–S2H). Tgm3 was not detected in any samples; however, Tgm2 levels specifically increased in SI5 mucus and peaked after 7-d WSD exposure (Figure 3C). Tgm2 transami-dates glutamine and lysine residues forming covalent isopeptide cross-links between polypeptides. We mined our data for isopeptide-linked Muc2 peptides and detected a cross-link between Muc2 Gln1047 and Lys1057 (Figure S2I). Detection of this cross-link increased in samples after 7-d WSD exposure, thus correlating with diet-dependent mucus dysfunction (Figure 3D). We subsequently tested the causal association of Tgm2 by feeding a WSD to *Tgm2*^−*/*−^ mice. While increased SI5 mucus penetrability was detected in WSD-fed littermate *Tgm2*^*+/+*^ mice, the same impact was not detected in WSD-fed *Tgm2*^−*/*−^ mice, thus indicating that WSD-induced SI5 mucus barrier dysfunction was Tgm2 dependent (Figures 3E and 3F).

Increased Muc2 cross-linking may affect mucus organization. To determine if WSD induced alterations in mucus layer structure, we employed a lectin-based approach for mucus visualization in live intestinal tissue.^22^ We validated this approach in CD-fed mice by staining SI5 mucus with a combination of fluorescently conjugated Ulex Europaeus Agglutinin I (UEAI) and Wheat Germ Agglutinin (WGA) lectins, and imaged lectins and tissue by confocal microscopy (Figure 3G). WGA stained the epithelial membrane, likely binding to the glycocalyx. Both UEA1 and WGA-stained material emerging from epithelial goblet cells, filling the spaces between intestinal villi (Figure 3G – yellow box). Higher-magnification imaging of UEA1/WGA-stained material revealed a continuous mesh (Figure 3G – orange box), corresponding with polymeric Muc2 network. We applied the same method to littermates fed a WSD for 7 d (Figure 3H) and observed a dramatic effect on mucus organization, with intervillus material becoming highly aggregated, resulting in large gaps in the overall mucus network (Figure 3H – purple box). Comparative quantification of mucus aggregation volume between revealed a significant increase in the WSD-fed group (Figures S3A and S3B). Aggregated mucus in CD-fed samples was largely limited to discrete material emerging from epithelial goblet cells, whereas continuous aggregates were observed in WSD-fed samples (Figure S3C).

This demonstrated that the SI5 mucus of CD-fed mice comprised a network of polymeric material secreted by epithelial goblet cells, which expanded and filled up the spaces between the intestinal villi. WSD exposure resulted in aggregated or unexpanded mucus that could not fill these spaces, resulting in loss of mucus barrier function independent of core proteome alterations. WSD induced increased Tgm2 abundance and Muc2 isopeptide cross-linking, which was causally linked to mucus dysfunction.

### Mucus barrier ablation permits atypical *Citrobacter rodentium* infection of the jejunum

Having established the basis of diet-induced SI5 mucus dysfunction, we sought to determine its consequences. Disruption of total intestinal mucus barrier function has been linked to increased susceptibility to colonic infection and microbiota-driven inflammation; however, the jejunum has been ignored in such studies. In agreement with prior studies,^23^ we found that infecting wild-type (WT) CD-fed mice with the enteric bacterial pathogen *C. rodentium* resulted in primary colonization of the distal colonic mucosa and high pathogen stool load, with limited colonization of small intestinal tissues and no detectable translocation into the mesenteric lymph nodes (MLNs) (Figure S4A). This colonization pattern may be influenced by differences in mucosal defenses between the small and large intestine, rather than a more specific tropism of *C*. *rodentium*, thus we hypothesized that dietary disruption of the SI5 mucus barrier could render this region more susceptible to pathogen colonization.

**Figure 4.**
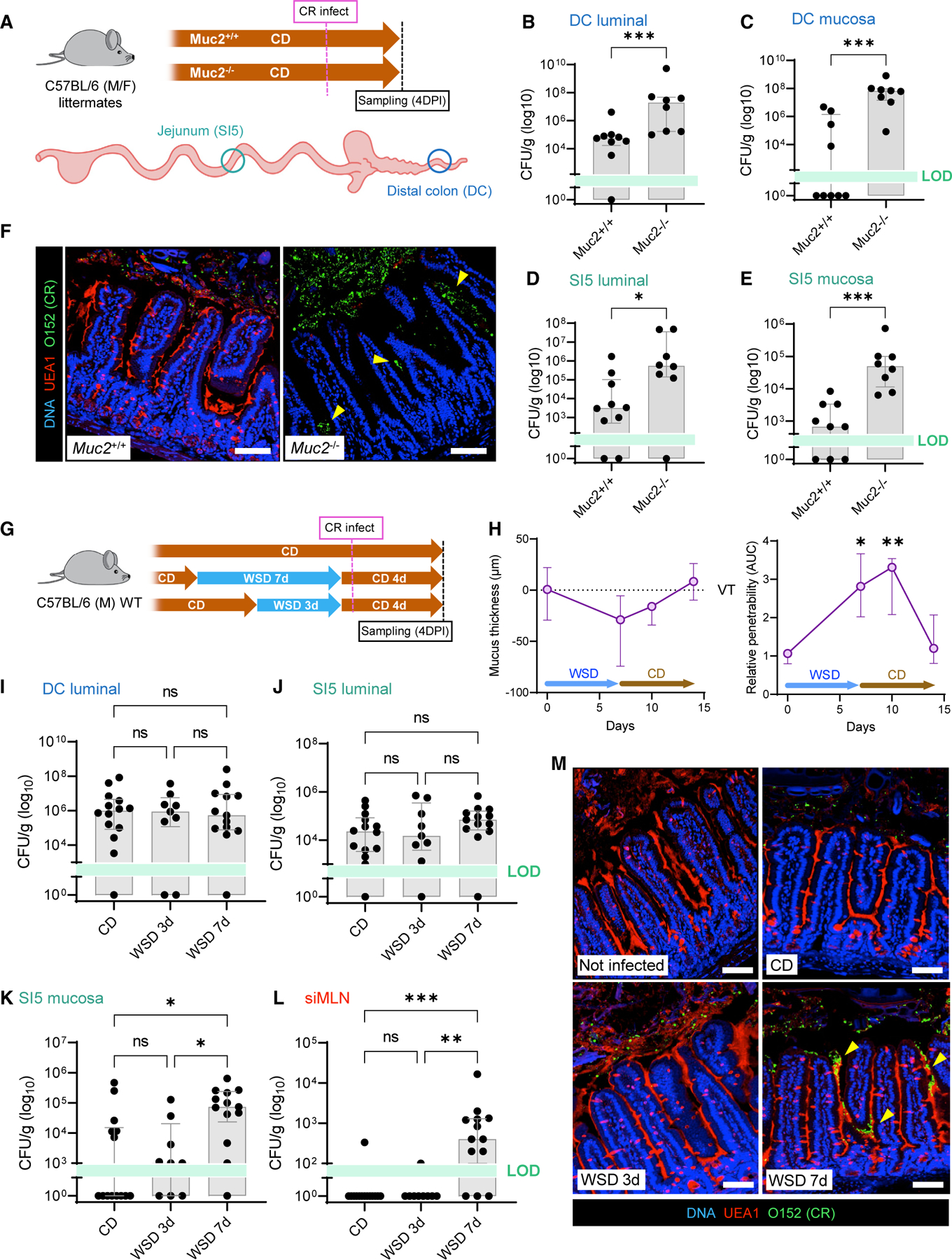
Genetic or diet-induced mucus disruption results in increased susceptibility to jejunal C. rodentium infection (A–F) Infection of CD-fed *Muc2*^*+/+*^ and *Muc2*^−*/*−^ littermate mice with *C*. *rodentium*. Schematic illustrating infection time course and intestinal tissue sampling points (A). *C*. *rodentium* colony-forming unit (CFU) enumeration from DC stool (B), DC mucosa (C), SI5 contents (D), and SI5 mucosa (E). Confocal micrographs of fixed SI5 tissue sections from *C*. *rodentium*-infected mice stained for DNA (blue), mucus (UEA1; red), and *C*. *rodentium* LPS (O152; green) (F). (G–M) Infection of CD, WSD 3 d, and WSD 7 d-fed WT mice with *C*. *rodentium*. Schematic illustrating dietary interventions and infection time course (G). Quantification of mucus thickness (left) relative to villus tips (VTs) and barrier function (right) in SI5 of mice fed WSD for 7 d and switched back to CD (H). *C*. *rodentium* CFU enumeration from DC stool (I), SI5 contents (J), SI5 mucosa (K), and siMLN (L). Confocal micrographs of fixed SI5 tissue sections from different experimental groups, stained as described for (F). All image scale bars are 50 µm. Data show median and interquartile range for n = 8–9 (B–E) and n = 9–14 (I–L) mice per group as indicated. Significance by Mann-Whitney (B–E) or Dunn’s multiple comparison (H–L) test (*p < 0.05, **p < 0.01, ***p < 0.001, ns: not significant). All infection experiments represent pooled data from two independent experiments, n = 4–7 mice per group per experiment. LOD, limit of detection.

To test this hypothesis, we infected CD-fed *Muc2*^*+/+*^ and *Muc2*^−*/*−^ littermates with *C*. *rodentium* to determine the effect of mucus loss on pathogen colonization (Figure 4A). *Muc2*^−/−^ mice develop colonic inflammation as they age, which may confound infection experiments; however, we detected no signs of jejunal inflammation in uninfected mice (Figure S4B). Infected mice were examined at 4 days post infection in order to focus on pathogen colonization, rather than host-response and clearance processes. As previously reported,^15^ *C*. *rodentium* load in both distal colon luminal and mucosa samples was significantly higher in *Muc2*^−*/*−^ compared with *Muc2*^*+/+*^ mice (Figures 4B and 4C). However, we also observed a similar difference in SI5 luminal and mucosal samples (Figures 4D and 4E), which was supported by immunohistochemical detection of intervillus and epithelium-associated *C*. *rodentium* in fixed SI5 tissue sections from *Muc2*^−*/*−^ mice (Figures 4F and S4C). Consequently, this supported the idea that disruption of the mucus layer allowed pathogen colonization of an atypical intestinal niche.

We next examined the functional consequence of diet-induced SI5 mucus barrier disruption on susceptibility to *C*. *rodentium* colonization. WT mice were fed a WSD for 3 d (impenetrable mucus) or 7 d (penetrable mucus) and compared with CD-fed controls (Figure 4G). WSD-fed groups were switched to CD 1 d prior to infection to discriminate the impact of the diet on the host, rather than any effect of WSD on *C*. *rodentium*. This was validated in a separate group, where we determined that WSD-induced SI5 barrier dysfunction persisted for a least 3 d after switching to CD (Figures 4H and S4D). Comparison of *C*. *rodentium* load in SI5 lumen, colonic lumen, and colonic mucosa detected no significant differences among CD, WSD 3 d, and WSD 7 d-fed groups (Figures 4I and 4J and S4E); however, a substantial and significant increase in SI5 mucosal load was observed in the WSD 7 d-fed group compared with both CD and WSD 3 d-fed mice (Figure 4K). Almost exclusive detection of *C*. *rodentium* in the small intestine-draining MLN (siMLN) in the WSD 7 d-fed group indicated that the pathogen was able to breach the small intestinal barrier in these mice (Figure 4L). *C*. *rodentium* load data were supported by staining of fixed SI5 tissue sections, which detected intervillus and epithelium-associated *C*. *rodentium* in WSD 7 d-fed mice (Figures 4M and S4F and S4G).

These data demonstrated that exposure to a WSD induced susceptibility to SI5 *C*. *rodentium* infection that coincided with loss of mucus barrier function. Accordingly, similar results from infection of mice that lack the key mucus structural component Muc2 supported the concept that disruption of the jejunal mucus barrier permits atypical colonization of this intestinal region by a pathogen that is primarily associated with the distal intestine under normal circumstances.

### Microbiota colonization of the jejunum is dependent on an intact mucus layer

Given the increased susceptibility to infection, we reasoned that diet-dependent SI5 mucus barrier dysfunction might result in an increase in the bacterial burden in the jejunum. We based this on our live tissue imaging data (Figures 3G and 3H), as we found that the mucus aggregates at the tips of the villi of non-infected WSD-fed mice were frequently associated with dense clusters of microbial cells (Figure S5A), thus indicating that diet-induced mucus aggregation might promote bacterial colonization and overgrowth in the small intestine.

**Figure 5.**
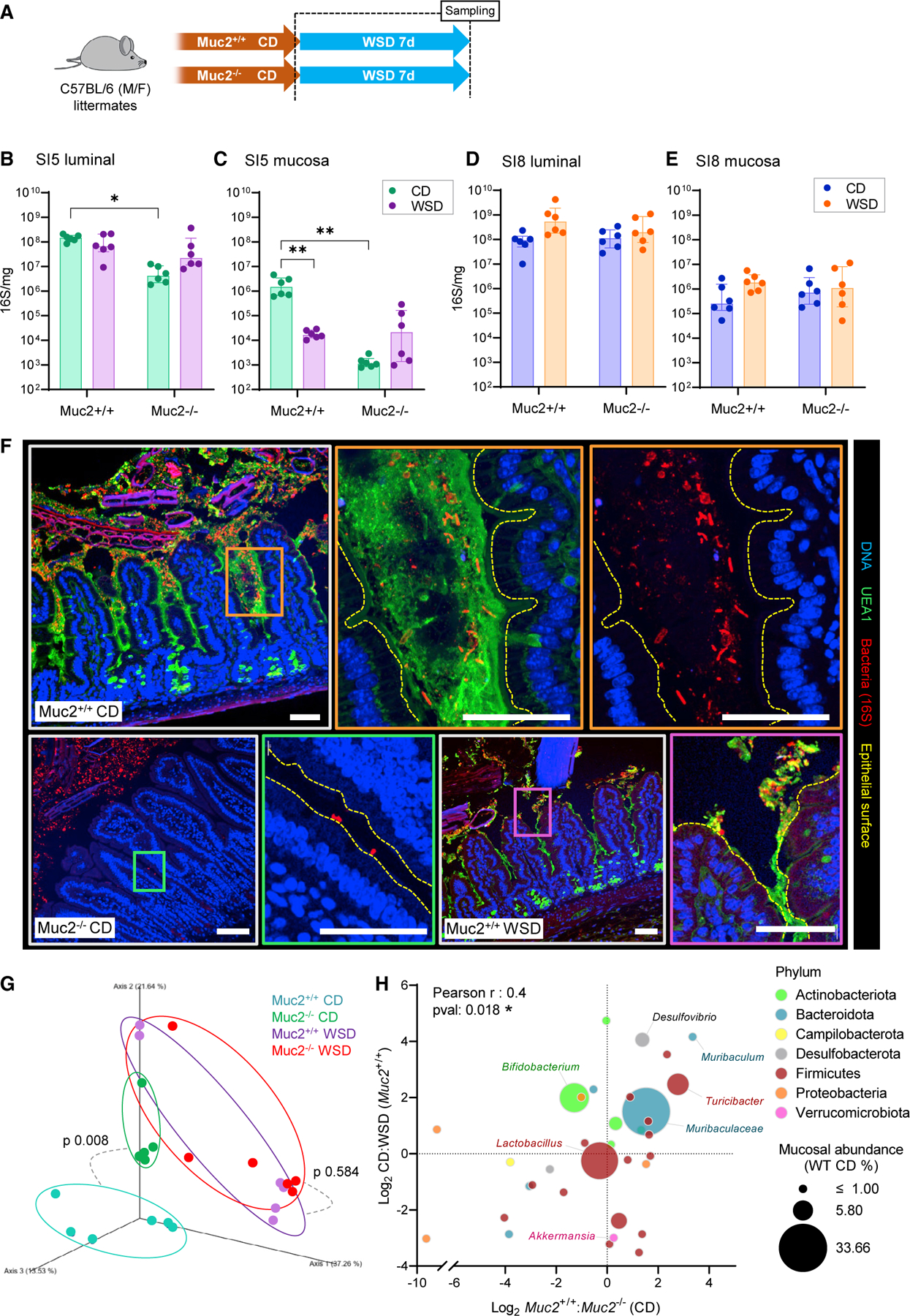
Microbiota colonization of the jejunal mucosa is dependent on an intact mucus layer (A–F) Quantification and localization of SI5 and SI8 microbiota in CD and WSD-fed *Muc2*^*+/+*^ and *Muc2*^−*/*−^ littermate mice. Schematic illustrating dietary interventions and sampling time points (B). Quantification of total bacterial load in SI5 contents (C), SI5 mucosa (D), SI8 contents (E), and SI8 mucosa (F) by 16S rRNA gene qPCR. Confocal micrographs of fixed SI5 tissue sections from different experimental groups stained to detect DNA (blue), mucus (UEA1; green), and bacteria (16S FISH; red) (G); higher-magnification images from *Muc2*^*+/+*^ CD-fed (orange panels), *Muc2*^−*/*−^ CD-fed (green panel), and *Muc2*^*+/+*^ WSD-fed (purple panel) mice are shown. (G and H) Analysis of microbiota community structure in SI5 mucosa of CD and WSD-fed *Muc2*^*+/+*^ and *Muc2*^−*/*−^ littermate mice by 16S rRNA gene sequencing. Principal-component analysis of β-diversity (Bray-Curtis dissimilarity) of the bacterial microbiota (G). Comparison of log_2_ CD:WSD (*Muc2*^*+/+*^) and *Muc2*^+/+^:*Muc2*^−/−^ (CD-fed) abundance ratios of bacterial taxa detected in all groups (H); data points represent individual taxa color coded by phylum and sized by median mucosal relative abundance (RA) in CD-fed *Muc2*^+/+^ mice. All image scale bars are 30 µm. Images are representative of n = 6 mice per group. Data show median and interquartile range for n = 6 (B–E) mice per group. Significance by Tukey test (*p < 0.05, **p < 0.01, ns: not significant). Experiments represent pooled data from two independent experiments, n = 3 mice per group per experiment.

To examine the interactive effect of diet and mucus on microbiota colonization of the small intestine, we quantified bacterial load in luminal and mucosal SI5 and SI8 samples from CD and WSD-fed *Muc2*^*+/+*^ and *Muc2*^−*/*−^ littermate mice (Figure 5A) by 16S rRNA gene qPCR (Figures 5B–5E). Surprisingly, 16S quantification revealed a 100-fold decrease in bacterial load in the mucosa of WSD-fed *Muc2*^*+/+*^ mice (Figures 5B and 5C), indicating that WSD suppressed mucosal colonization. Furthermore, we observed that CD-fed *Muc2*^−/−^ mice had significantly reduced bacterial density in the lumen (25-fold) and mucosa (>1000-fold) compared with their *Muc2*^+/+^ littermates, and that this was not further altered by WSD exposure (Figures 5B and 5C). Microbiota suppression by both WSD and Muc2 deficiency in SI5 was regiospecific, as we detected no dietor genotype-dependent effects on bacterial density in either luminal or mucosal SI8 samples (Figures 5D and 5E).

Our data demonstrated that the SI5, but not SI8, mucosal microbiota was dependent on the presence of Muc2. These findings potentially linked the microbiota depletion driven by WSD exposure to SI5 mucus layer disruption, as WSD exposure of *Muc2*^−*/*−^ mice had no additive effect on bacterial load. This indicated the existence of a mucosal SI5 microbiota that was dependent on the presence of an intact jejunal mucus layer. The presence of mucus-associated bacteria was verified by 16S rRNA fluorescence *in situ* hybridization (FISH) in fixed *Muc2*^*+/+*^ SI5 tissue, which detected bacterial cells in mucus between the intestinal villi (Figure 5F). Notably, mucus-associated bacteria were not in contact with epithelial cells, indicating that functional separation was maintained despite high proximity. In accordance with qPCR data, imaging bacteria in tissue sections from both *Muc2*^−/−^ and WSD-fed *Muc2*^*+/+*^ mice detected almost no bacteria between the intestinal villi, and those that were present were often in direct contact with epithelial cells or found in mucus aggregates at the villus tips (Figure 5F– purple box).

As both WSD and *Muc2* knockout had a similar effect on bacterial load and distribution, we hypothesized that they might also have similar effects on mucosal microbiota community structure. Comparison of microbiota β-diversity found that *Muc2* deficiency had a significant impact in CD, but not WSD-fed mice (Figure 5G). Notably, the microbiota of CD-fed *Muc2*^−/−^ mice clustered closer to WSD-fed mice compared with CD-fed *Muc2*^+/+^ controls. Comparison of the effect of WSD and *Muc2* deficiency on taxon abundances identified some taxa (e.g., *Bifidobacteria* and *Akkermansia*) that were primarily affected by diet but not *Muc2* deletion. However, the impact on the majority of taxa was similar, resulting in a significant positive correlation between WSD and *Muc2* knockout effects (Figure 5H). Conversely, comparative analysis of the impact of WSD exposure in *Muc2*^*+/+*^ and *Muc2*^−/−^ mice detected no significant correlation in taxon abundance (Figure S5B), indicating that the impact of WSD exposure on microbiota composition was influenced by the presence or absence of Muc2.

Thus we identified a striking similarity between WSD exposure and *Muc2* deficiency on SI5 microbiota load, distribution, and community structure. Both factors result in loss of the expanded mucus network that serves as habitat of the mucosa-associated microbiota. Consequently, our data supported the existence of a mucosal microbiota that was dependent on the presence of an intact mucus layer, and that was highly sensitive to diet-induced loss of the jejunal mucus niche.

### Muc2-dependency correlates with bacterial lifestyle and reduced environmental antimicrobial potential

**Figure 6.**
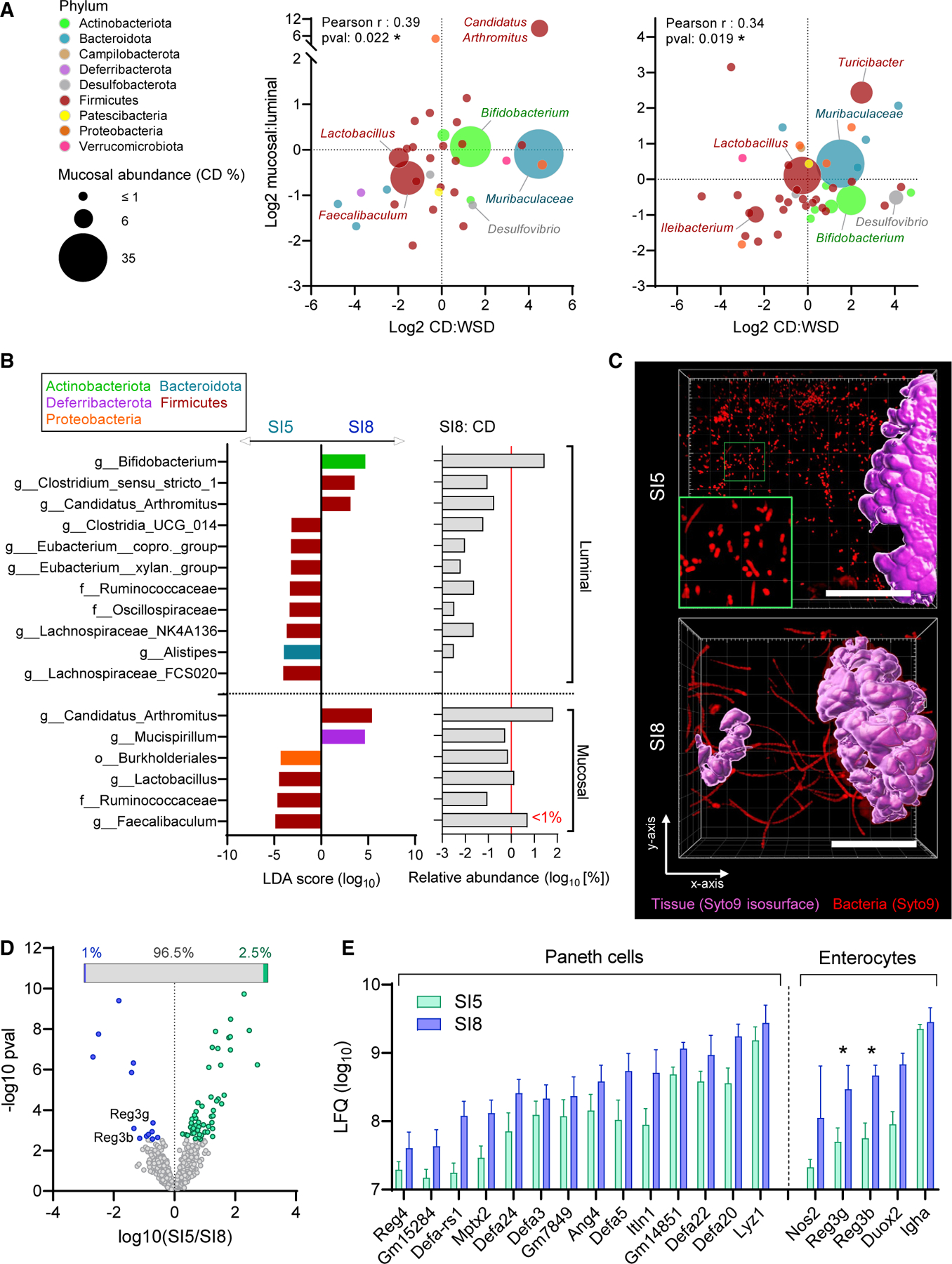
Microbial and host factors in the jejunal and ileal mucus niche (A) Comparison of log_2_ mucosal:luminal and CD:WSD ratios for bacterial taxa detected in SI5 mucosal samples of CD and WSD-fed mice by 16S rRNA gene sequencing. Panels show data from independent experiments detailed in Figure 2 (left) and Figure 5 (right); data points represent individual taxa color coded by phylum and sized by median mucosal relative abundance (RA) in CD-fed mice. (B) Linear discriminant analysis (LDA) effect size identification of bacterial taxa significantly enriched in SI5 or SI8 luminal and mucosal microbiota (left panel) aligned with the relative abundance of bacterial taxa in SI8 of CD-fed mice (right panel). Red line indicates a relative abundance of 1%. (C) *Ex vivo* confocal microscopy imaging of tissue (purple isosurface) and bacteria (red) in SI5 and SI8 of CD-fed mice. Images are confocal z stacks showing x/y axis projections and a magnified image of planktonic bacterial cells in SI5 (green panel). (D and E) Mass spectrometry-based label-free quantification (LFQ) and comparison of SI5 and SI8 mucus proteomes from CD-fed mice. Volcano plot illustrating proteins significantly more abundant in either SI5 or SI8 (D); inset shows proportion of discriminant proteins as a percentage of all detected proteins. Abundances of all detected Paneth cell and enterocyte-specific antimicrobial proteins (E). All image scale bars are 40 µm. Data show average values from n = 5–6 mice per group (A) or median and interquartile range for n = 8 mice per group with significance by t test and Permutation-based false discovery rate (*p < 0.05) (D, E).

We subsequently sought to identify factors that might promote the role of jejunal mucus as a microbial habitat. We first examined our 16S rRNA gene sequencing data from two independent experiments (Figures 2 and 5) in order to assess SI5 mucosal microbiota community structure, luminal-mucosal distribution, and sensitivity to WSD-induced mucus disruption (Figure 6A). This demonstrated that bacterial taxa that dominated the mucosal environment (Muribaculaceae, *Bifidobacterium*, *Faecalibaculum,* and *Lactobacillus*) were also abundant in the luminal environment, indicating continuous interaction between the two compartments. Conversely, compartment-enriched genera such as luminal Streptococcaceae or mucosal Candidatus Arthromitus (segmented filamentous bacteria: SFB) were minor components of the SI5 community (Figure 6A). There was a significant positive correlation between specific taxa mucosal:luminal and CD:WSD-fed abundance ratios, indicating that enrichment in the mucosal compartment correlated with increased susceptibility to suppression by WSD.

We hypothesized that differences in microbiota community structure may account for variability in Muc2-dependency between SI5 and SI8. Comparative analysis of 16S sequencing data from SI5 and SI8 samples by LEfSe identified SFB as one of a limited number of taxa significantly enriched in the luminal and mucosal environments in SI8 compared with SI5 (Figure 6B). SFB abundance was particularly high in the SI8 mucosal environment where it was the dominant bacterial taxon. Imaging data indicated that bacteria in SI5 mucus existed as planktonic cells (Figure 6C), whereas SFB colonizing SI8 existed as epithelium-associated filaments (Figures 6C and S6A). Accordingly, the lifestyle of the SI5 and SI8 mucosal microbiota co-varied with niche-specific Muc2-dependency.

Secretion of antimicrobial peptides (AMPs) by Paneth cells and enterocytes is a factor that controls the capacity of the microbiota to colonize the small intestinal mucus layer.^9,24^ We therefore analyzed the SI5 and SI8 mucus proteome (Figure 6D and Table S3) and found the majority of proteins (96.5%) were similarly detected in both environments. Multiple Paneth cell and enterocyte-derived AMPs were detected in both SI5 and SI8 mucus, and the enterocyte-derived AMPs Reg3g and Reg3b were detected at significantly lower level in SI5 compared with SI8 mucus (Figures 6D and 6E). We last examined how the microbiota influences the overall antimicrobial landscape in SI5 and SI8 by mining our previous dataset^25^ quantifying the intestinal epithelial proteome of conventionally raised (ConvR) and germ-free (GF) mice (Figure S5B). In line with previous investigations,^26^ expression of Reg3b and Reg3g was microbiota dependent in both regions, suggesting that the differential detection of these proteins in SI5 and SI8 mucus was determined by varying degrees of microbial induction.

Our data demonstrated that jejunal and ileal mucus represent highly divergent environments. Ileal mucus was differentiated by high abundance of enterocyte-derived AMPs and colonization by epithelium-embedded SFB. Conversely, jejunal mucus had lower antimicrobial potential, and was colonized by planktonic bacterial cells from more diverse taxa. These data indicated that the variable intrinsic properties of the jejunal and ileal mucus layers are likely to be causally linked to the establishment of microbial communities with varying dependence on Muc2.

### The jejunal microbiota confers resistance to atypical *C. rodentium* infection

The microbiota can provide colonization resistance against enteric pathogen colonization. Given that both WSD exposure and deletion of Muc2 resulted in concurrent loss of the jejunal mucus barrier function and Muc2-dependent bacteria, this suggested that increased susceptibility to jejunal *C*. *rodentium* infection might be due to disruption of colonization resistance rather than as a direct loss of mucus barrier function.

**Figure 7.**
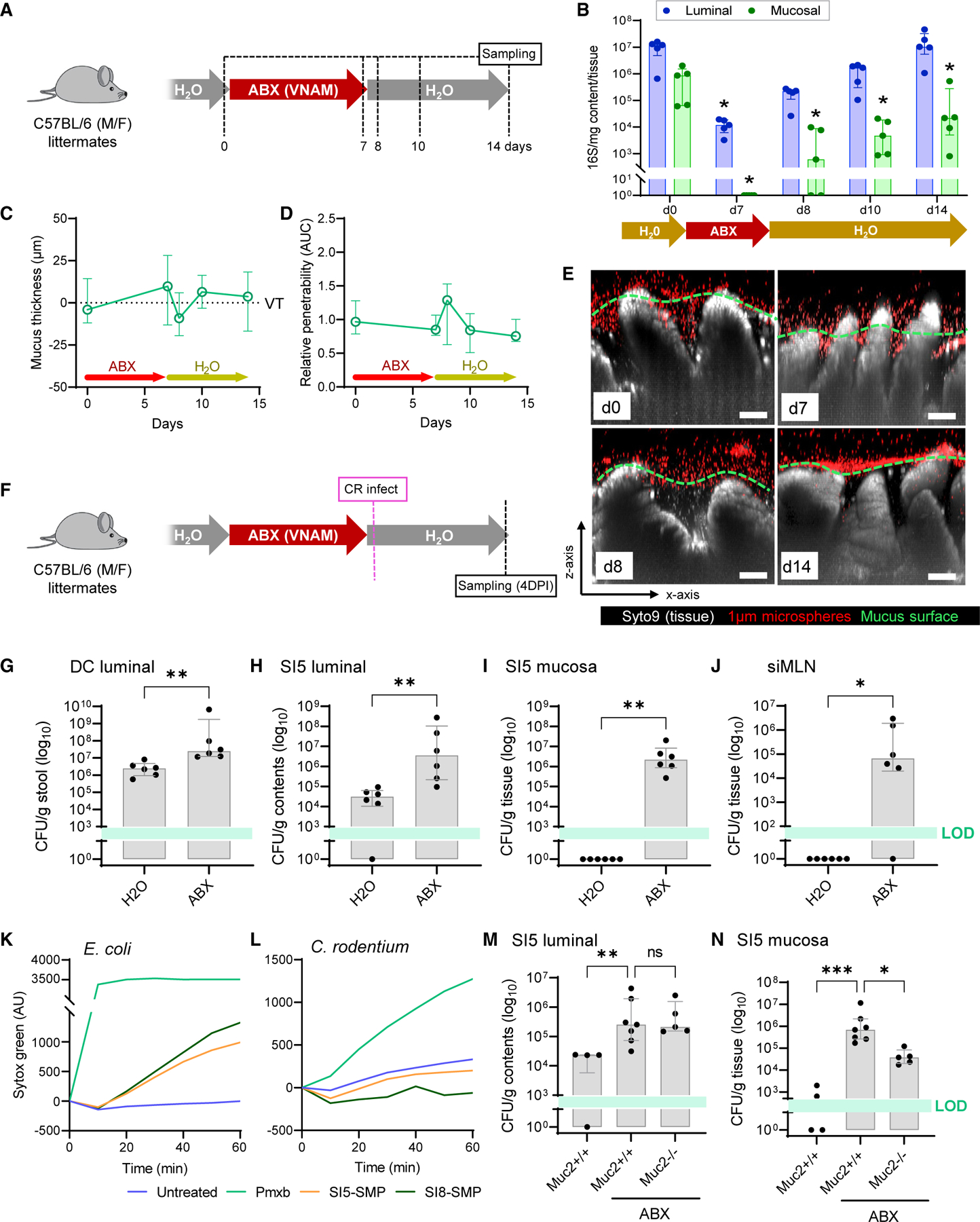
The jejunal microbiota mediates susceptibility to *C. rodentium* (A–E) Jejunal microbiota depletion using broad-spectrum antibiotics (ABX). Schematic illustrating ABX treatment schedule and sampling points (A). Quantification of SI5 luminal and mucosal bacterial load by 16S qPCR (B). Quantification of SI5 mucus thickness (C) relative to villus tips (VTs) and relative barrier function (D) based on data extracted from images shown in (E). Confocal z stacks showing x/z axis cross sections of *ex vivo* SI5 tissue (gray) and 1-µm microspheres (red) from mice before and after ABX treatment (E); approximate mucus surface (green dashed line) indicated. Data in (D) are normalized to day 0 group mean. (F–J) Infection of ABX-treated mice with *C*. *rodentium*. Schematic illustrating ABX treatment, infection, and sampling schedule (F). *C*. *rodentium* CFU enumeration from DC stool (G), SI5 contents (H), SI5 mucosa (I), and siMLN (J). (K and L) Permeabilization of *E*. *coli* (K) or *C*. *rodentium* (L) cell suspensions by Polymyxin B (Pmxb) or soluble mucus protein (SMP) prepared from SI5 or SI8 mucus. Data representative of two independent experiments. (M and N) Infection of ABX-treated *Muc2*^+/+^ and *Muc2*^−/−^ mice with *C*. *rodentium*. CFU enumeration from SI5 contents (M) and SI5 mucosa (N). All image scale bars are 50 µm. Images are representative of n = 5 mice per group. Data show median and interquartile range for n = 5 (B–D), n = 6 (G–J) or n = 4–6 mice per group as indicated. Significance by Dunnet (B–D), Mann-Whitney (G–J), or Dunn’s (M, N) test (*p < 0.05, **p < 0.01, ***p < 0.0001). All infection experiments represent pooled data from two independent experiments, n = 2–3 mice per group per experiment. LOD, limit of detection.

To differentiate the roles of colonization resistance and mucus barrier function in jejunal infection, we sought to deplete the microbiota while maintaining SI5 mucus barrier integrity by treating CD-fed mice with ABX. We validated this approach by exposing mice to ABX for 7 d and assessing SI5 bacterial load and mucus barrier properties (Figure 7A). ABX treatment suppressed luminal bacterial load (1,000-fold) and reduced the mucosal bacterial load to undetectable levels (Figure 7B). Post-ABX treatment, bacterial load gradually recovered; however, mucosal load remained significantly reduced up to 7 d post-ABX exposure. Quantification of SI5 mucus barrier properties over the same period found no effect of ABX exposure on either SI5 mucus thickness or barrier function (Figures 7C–7E).

Mice were subsequently exposed to ABX for 7 d to deplete the microbiota, switched to normal drinking water, and then infected with *C*. *rodentium* (Figure 7F). Comparison of *C*. *rodentium* burden in ABX-treated mice with control mice indicated that ABX treatment resulted in higher luminal *C*. *rodentium* load in stool (Figure 6G), indicating that microbiota depletion enhanced *C*. *rodentium* colonization in the pathogen’s typical niche. However, ABX treatment also resulted in a substantial increase in *C*. *rodentium* load in the SI5 content and mucosal samples (Figures 6H and 6I), thus demonstrating that depletion of the microbiota induced susceptibility to atypical jejunal *C*. *rodentium* colonization. Importantly, we exclusively detected live *C*. *rodentium* in siMLN samples from the ABX-treated mice (Figure 6J), indicating that microbiota depletion also allowed the pathogen to breach the small intestinal barrier.

Colonization resistance may be conferred by the microbiota via several mechanisms, including production of antimicrobial factors or by occupation of the colonization niche. In order to determine if small intestinal mucus contained antimicrobial factors that might target *C*. *rodentium*, we treated cultures with soluble mucus proteins (SMP) from both SI5 and SI8 and quantified bacterial membrane permeabilization. Both *E*. *coli* and *C*. *rodentium* cells were efficiently permeabilized by the antibiotic Polymyxin B; however, SMP from either SI5 or SI8 was only able to permeabilize *E*. *coli* and had no detectable effect on *C*. *rodentium* membrane integrity (Figures 7K and 7L), thus indicating that *C*. *rodentium* is resistant to microbiota antimicrobial factors found in our SMP preparations. We investigated the potential role of the microbiota in blocking colonization of the jejunal mucus niche by examining the colonization of *C*. *rodentium* in the context of ABX-mediated microbiota depletion in *Muc2*^+/+^ and *Muc2*^−/−^ littermates. While no difference was detected in SI5 luminal *C*. *rodentium* load between ABX-treated *Muc2*^+/+^ and *Muc2*^−/−^ samples (Figure 7M), the pathogen burden in SI5 mucosal samples was significantly (>10 fold) higher in ABX-treated *Muc2*^+/+^ compared with ABX-treated *Muc2*^−/−^ samples (Figure 7N), indicating that mucus promotes *C*. *rodentium* colonization in the absence of an intact microbiota.

Our data thus demonstrated that resistance to atypical jejunal colonization by *C*. *rodentium* was functionally dependent on the microbiota. Rather than acting as a barrier to colonization, jejunal mucus promotes pathogen colonization unless it is occupied by the microbiota. As *C*. *rodentium* appeared to be resistant to antimicrobial factors present in the jejunal mucus, these findings suggest that the microbiota passively generates colonization resistance by occupying the jejunal mucus niche.

## DISCUSSION

Mucus barrier systems have developed to cope with microbial challenges by segregating them from the epithelial surface, maintaining a balance between the host and microbiota. Homeostasis has evolved in the context of a varied diet, low in fat and rich in complex polysaccharides, and evidence suggests that a WSD can destabilize this relationship.^27,28^ While previous investigations have focused on the colon, we have now determined that WSD exposure has a deleterious effect on jejunal mucus that sensitizes to bacterial infection. Crucially, our data indicate that local resistance to infection is mediated by the presence of a Muc2-dependent microbiota that provides colonization resistance against the pathogen. Consequently, these findings identify a novel mechanism by which the host actively supports colonization resistance, and illustrates that this symbiotic relationship is sensitive to dietary disruption.

The concept of mucus as a defensive structure has driven investigations that characterize intestinal mucus layers in relation to their barrier function.^6,8,11,12,29^ Mucus specialists such as *Helicobacter*, *Mucispirillum,* and SFB colonize niches within the GI mucus that are inaccessible to other microbes; however, the colonic and ileal mucus layers remain largely devoid of microbes under normal conditions. Conversely, the Muc2 dependency of the jejunal microbiota and images revealing bacterial cells within the mucus layer (see Figure 5), indicate that jejunal mucus functions as a habitat rather than a barrier. It is not evident how the bacterial taxa that dominate this niche have evolved to do so in the absence of flagella or epithelial attachment mechanisms; however, it is notable that enterocyte-derived antimicrobials are low in jejunal compared with ileal mucus (Figure 6E), as *Bifidobacterium longum* has previously been shown to suppress Reg3g expression,^30^ suggesting that microbiota-host communication may play a role in tuning the jejunal mucus habitat.

Our findings highlight a novel interaction among diet, mucus, and colonization resistance. Prior studies targeting the influence of WSD or low-fiber diets on the colonic mucus barrier or colonization resistance against *C*. *rodentium* consistently demonstrate causal links to the microbiota.^11,12,31,32^ Our data now illustrate WSD-driven jejunal mucus layer collapse, disrupting an important microbiota habitat and thereby negating local colonization resistance. It has been speculated that intestinal mucus might support colonization resistance^33,34^; however, the barrier function of colonic mucus complicates analysis of its role in colonization resistance due to the pleiotropic effects of *Muc2* knockout on colonic host-microbiota interactions. The fact that jejunal mucus functions as a microbial habitat, as opposed to a barrier, removes this complication and now allows us to define mucus as a host factor that actively supports colonization resistance against *C*. *rodentium*.

While the causal role of the microbiota in WSD-driven colonic mucus barrier dysfunction is established, its role in the jejunum is less clear cut. Antibiotic depletion of the microbiota prior to WSD exposure did not prevent its impact on mucus properties, suggesting that the effects of WSD are dysbiosis-independent. However, failure to successfully re-engraft the normal microbiota in WSD-fed mice leaves this open to doubt. Nevertheless, the resistance of the WSD-fed jejunum to recolonization is logically consistent with the finding that the normal jejunal microbiota is dependent on an intact mucus layer.

Enteric bacterial infections are relatively rare in developed regions; however, antibiotic use, diabetes, and obesity are all risk factors for small intestinal bacterial overgrowth (SIBO), a condition that is characterized by aberrant expansion of bacteria in the small intestine that can result in chronic diarrhea and malabsorption,^35,36^ which may be functionally linked to loss of colonization resistance. We may speculate that the expansion of Western dietary habits into regions where the risk of enteric bacterial infections is higher may result in exposure of an increasing number of individuals with reduced jejunal colonization resistance to pathogens that can exploit such deficiencies.

### Limitations of the study

We have focused our analyses on the impact of several different experimental interventions (e.g., diet, *C*. *rodentium* infection, ABX, gene knockout) on the small intestine; however, it should be noted that all have an impact along the entire intestine. Our experiments allow us to make specific conclusions regarding their effects on host-microbiota interactions that we have quantified in the small intestinal environment. However, current inability to selectively deplete the small intestinal microbiota or regiospecific conditional gene knockout models mean that it is not possible to isolate the specific impact of jejunal microbiota colonization on overall host health.

## STAR★METHODS

### RESOURCE AVAILABILITY

#### Lead contact

Information and requests for resources and reagents should be directed to and will be fulfilled by the lead contact, George Birchenough (george.birchenough@gu.se).

#### Materials availability

This study did not generate new unique reagents.

#### Data and code availability

- Data availability: Mass spectrometry proteomics data have been deposited to the ProteomeXchange Consortium via the PRIDEpartner repository with the dataset identifier ProteomeXchange: PXD028613. Microbiota 16S rDNA gene sequencing results have been deposited in the ENA sequence read archive with accession number ENA: PRJEB47610.
- Code availability: This paper does not report original code.
- Any additional information required to reanalyze the data reported in this paper is available from the lead contact upon request.

### EXPERIMENTAL MODEL AND SUBJECT DETAILS

All mice used in experiments were either bred in-house or purchased from Charles River (Germany). Purchased mice were facility-acclimatized for 3 weeks in cages containing used bedding from in-house bred mice. All mice were on a C57BL/6 background and were housed under specific pathogen free conditions with *ad libitum* access to food and water with a 12h light/dark cycle. Experimental groups consisted of age-matched 12–17 week old male mice, or a balanced mixture of male and female mice as indicated in the figures for each experiment. Mice were fed a standard low fat, low sugar, high fiber chow diet (5021, LabDiet) or a high fat, high sugar, low fiber Western-style diet (TD.96132, Envigo). *Muc2* and *Tgm2* knockout mice have been previously described,^17,38^ and littermated experimental mice were generated using heterozygous breeding pairs. For antibiotic treatment experiments, vancomycin (0.5 mg/mL), neomycin (1 mg/mL), ampicillin (1 mg/mL) and metronidazole (1 mg/mL) were purchased from Merck and dissolved in drinking containing 1% w/v sucrose at the indicated concentrations. Animals were anesthetized using isoflurane and killed by cervical dislocation before collection of samples. All experimental procedures involving animals were approved by the Swedish Laboratory Animal Ethical Committee in Gothenburg.

### METHOD DETAILS

#### *Ex vivo* quantification of small intestinal mucus barrier properties

Determination of small intestinal mucus thickness and barrier function was adapted from a previous *ex vivo* method used to study similar properties in colonic tissue.^18^ Briefly, approximately 3 cm tissue from different small intestinal regions was flushed with ice-cold oxygenated Krebs buffer to remove luminal content, opened longitudinally and mounted in a horizontal perfusion chamber as previously detailed.^50^ Tissue was overlaid with Krebs buffer containing a mixture of Syto9 cell dye (25 µm, ThermoFisher) and 1 µm crimson carboxylate-modified Fluospheres microbeads (1:20 dilution, ThermoFisher) and incubated for 15 min. The tissue was then washed with 0.5 mL Krebs buffer then submerged in 2 mL fresh Krebs buffer for imaging.

Tissue and microbeads were imaged using an LSM700 laser scanning confocal microscope equipped with an ×20 water-immersion objective, 488/639-nm lasers, and Zen acquisition software (Carl Zeiss). Tissue (small intestinal villi) and microbead fluorescent signals were mapped using Imaris software (Oxford Instruments) and data describing the z axis position of individual villus tips and microbeads was extracted. Mucus layer thickness was quantified in relation to villus tips by calculating average villus tip-microbead z axis distance. Mucus barrier function (normalised penetrability) was quantified by analysis of microbead distribution within the mucus layer. A frequency distribution curve of microbead z axis distance from the base of the small intestinal villi was generated for each z stack using Prism 9 software (GraphPad). Curves were normalized to maximum frequency values and then normalized to the position of the mucus surface and cropped to exclude data from microbeads above the mucus surface. Lastly, we generated area under the curve data expressed as normalized penetrability in order to allow quantitative comparison of microbead penetration into the mucus layers of different samples.

#### Intestinal microbiota profiling by 16S rRNA gene sequencing

DNA from intestinal content and mucosal tissue was extracted by mechanical lysis using a Fast-Prep System with Lysing Matrix E tubes (MPBio) as previously described.^51^ Bacterial microbiota composition was profiled by sequencing of the V4 region of the 16S rRNA gene on an Illumina MiSeq (Illumina RTA version 1.17.28; MCS version 2.5) using 515F and 806R dual indexing primers^39^ and the V2 kit (2 × 250 bp paired-end reads). Content samples were amplified in duplicate and mucosal samples were amplified in triplicate 25 µL reactions containing Five Prime Hot Master Mix (Quantabio), primers (200 nM), BSA (0.4 mg/mL), DMSO (5% v/v), and 20 ng (content) or 100 ng (mucosal) of DNA. PCR conditions were denaturation for 3 min at 94°C, followed by 25 cycles (content) or 26 cycles (mucosal) of denaturation for 45 s at 94°C, annealing for 60 s at 52°C, and elongation for 90 s at 72°C, and a final elongation step for 10 min at 72°C. Replicates were pooled then purified with NucleoSpin Gel and PCR Clean-up kit (Macherey-Nagel) and quantified using Quant-iT PicoGreen dsDNA kit (ThermoFisher). Equal amounts of purified PCR products were pooled and were purified again using Ampure magnetic purification beads (Agencourt) to remove short amplification products prior to sequencing.

Microbiota bioinformatics were performed with QIIME 2 2020.11.^41^ Raw sequence data were demultiplexed and quality filtered followed by denoising with DADA2.^42^ All amplicon sequence variants (ASVs) were aligned with mafft v.7.407^43^ and used to construct a phylogeny with fastTree v.2.1.10.^44^ Alpha-diversity metrics (Shannon diversity index H), beta diversity metrics (Bray-Curtis dissimilarity) and Principle Coordinate Analysis (PCoA) were estimated using the diversity core-metrics-phylogenetic command. Taxonomy was assigned to ASVs using the q2-feature-classifier^45^ classify-sklearn naïve Bayes taxonomy classifier against the Silva v.138 reference sequence database.^37^ Genus-level relative abundance data were correlated to different experimental groups using the LEfSe algorithm.^46^

#### Mass spectrometry-based profiling of the mucus proteome

Samples were collected *ex vivo* from intestinal tissues mounted in horizontal perfusion chambers as described above. Mucus was aspirated form the mucosal surface using Maximum Recovery pipette tips (Axygen), mixed with 2x cOmplete protease inhibitor cocktail (Merck) and stored at −80°C until analysis.

Sample processing was performed as previously described.^12^ Briefly, mucus was reduced overnight in 6 M guanidinum hydrochloride, 0.1 M Tris/HCl (pH 8.5), 5 mM EDTA, 0.1 M DTT (Merck) followed by filter aided sample preparation adapted from a previously developed protocol^52^ using 10 kDa cut-off filters (Pall Life Sciences). Proteins were alkylated with iodoacetamide (Merck) and sequentially digested with LysC (Wako) and trypsin (Promega) on the filter. Peptides were cleaned with StageTip C18 columns prior to MS analysis.^53^ NanoLC–MS/MS was performed on an EASY-nLC 1000 system (ThermoFisher), connected to a QExactive Hybrid Quadrupole-Orbitrap Mass Spectrometer (ThermoFisher) *via* a nanoelectrospray ion source. Peptides were separated using an in-house packed reverse-phase C18 column with a 60-min 4–32% acetonitrile gradient. Mass spectra were acquired from 320–1,600 m/z at resolution 70,000, and the 12 peaks with highest intensity were fragmented to acquire the tandem mass spectrum with a resolution of 35,000 and using automatic dynamic exclusion.

Proteins were identified using MaxQuant (v1.5.7.4)^47^ searching the mouse UniProt protein database (downloaded supplemented with mouse mucin sequences (http://www.medkem.gu.se/mucinbiology/databases/). Searches used full tryptic specificity, maximum 2 missed cleavages, 20 ppm precursor tolerance for recalibration search followed by 7 ppm for the final search, and 0.5 Da for fragment ions. Modifications were set as carbamidomethylation of cysteine (fixed), methionine oxidation (variable) and protein N-terminal (variable). The FDR was set to 1% both for peptide and protein levels and minimum peptide length was set to 6 amino acids. Proteins were quantified using label-free quantification (LFQ) using at least two peptides for quantification.

LFQ data was analyzed using Perseus (v1.6.2.2).^48^ Proteins were filtered for potential contaminants and detection in at least 50% of samples. Data was log_10_ transformed and missing values were imputed from a normal distribution using default settings. Two-sample tests (Student’s t-test or Welch’s t test) with Permutation-based FDR were used to identify specific protein abundance differences between experimental groups. Principal component analysis (PCA) was used to visualise clustering of different sample groups and similarity between groups were determined using PERMANOVA and Bray-Curtis dissimilarity methods in the Vegan package (v2.5–7) run in R (v4.1.1).

#### Detection of isopeptide cross-linked Muc2 peptides

Analysis of MS data for isopeptide cross-linked peptides was performed as previously described.^54^ Briefly, Mascot generic files (mgf) were searched against theoretical isopeptide crosslinks in murine MUC2 with the StavroX engine (version 3.6.6).^49^ Searches used full tryptic specificity and a maximum of 3 missed cleavages. Carbamidomethylation of cysteine was set as a fixed modification, Gln and Lys were set as cross-linking sites and the composition of the cross-linker was set to –NH3/-17.03 Da. Error tolerances of the parent ion and fragment ions were set to 2 ppm and 30 ppm respectively. The generated spectra were subsequently manually evaluated.

#### *Ex vivo* imaging of bacteria and mucus structure

Small intestinal tissues were mounted in horizontal perfusion chambers described above. Intestinal epithelial cells, mucus-associated bacterial cells and mucus were stained using Krebs buffer supplemented with 25 µm Syto9 cell dye (ThermoFisher), 50 µg/mL Ulex Europaeus Agglutinin I (UEA1)-DyLight649 conjugated lectin (Vectorlabs) and 50 µg/mL Wheat Germ Agglutinin (WGA)-Rhodamine conjugated lectin (Vectorlabs) for 15 min. Tissues were washed with 0.5 mL Krebs buffer then submerged in 2 mL fresh Krebs buffer for imaging. Epithelial cells, bacterial cells and lectin-bound mucus were imaged using an LSM700 laser scanning confocal microscope equipped with an ×20 water-immersion objective, 488/555/639-nm lasers, and Zen acquisition software (Carl Zeiss). In order to quantify mucus condensation in jejunal samples, Imaris software (Bitplane) was used to map isosurfaces to UEA1 signal based on a threshold level determined by first analysing images from small intestine of chow diet-fed mice. Identical isosurface mapping parameters were then applied to images from other experimental groups, and data describing the total isosurface volume and number of discrete isosurfaces was extracted. Imaging of bacterial cells in the mucus was conducted by acquiring high magnification and high resolution confocal z-stacks of areas where Syto9-stained bacterial cells were easily distinguishable form Syto9-stained epithelial cells. Images were processed in Imaris to distinguish bacterial and epithelial cells by mapping isosurfaces to Syto9 fluorescence using a manually defined threshold intensity that mapped high (epithelial cells) but not low (bacterial cells) fluorescence signals.

#### Infection of mice with Citrobacter rodentium

The nalidixic acid-resistant *C*. *rodentium* strain ICC169 (O152 serotype) was used for all infection experiments. Infection inocula were prepared by growing bacteria overnight in LB broth at 37°C in a rotating incubator. Overnight cultures were concentrated 10-fold by centrifugation at 4000 RCF for 10 min and resuspension in LB broth. Mice were gavaged with 200 μL of infection inoculum (1–3 × 10^9^ CFU). *C*. *rodentium* load at different anatomical sites was determined at specific time-points post infection by sacrificing mice and collecting samples under aseptic conditions. Approximately 3 cm jejunal, ileal and distal colonic tissues were dissected and flushed with 4 mL sterile PBS. Segment content and flushed tissue were collected separately. *C*. *rodentium* load in intestine-draining lymphatic structures was examined by sampling mesenteric lymph nodes. Depending on the experiment, MLNs were either sampled en masse or carefully separated into the nodes draining the small intestine (siMLN) and the node draining the caecum and proximal colon. All samples were homogenized in sterile PBS using an Ultra-Turrax T10 dispersing instrument (IKA) that was sequentially cleaned in 70% ethanol (×2) and sterile PBS. *C*. *rodentium* was enumerated from homogenates by serial dilution on Macconkey agar supplemented with 10 µg/mL nalidixic acid, followed by overnight incubation at 37°C and quantification of bacterial CFUs. For each sample, a theoretical limit of detection (LOD) was calculated based on detection of one colony at the lowest plated dilution. Average LOD calculated for all samples of the same type for each experiment is shown on all CFU graphs.

#### Histology

Intestinal tissues containing luminal content were fixed by submersion in methanol-Carnoy solution for at least 24 h, and fixed tissue was paraffin embedded and cut into 5 µm thick longitudinal sections. Tissue sections were deparaffinised by sequential washing in xylene substitute (20 min at 60°C; Merck) and 100% (5 min), 95% (5 min), 70% (5 min), and 30% (5 min) ethanol. For histochemical staining, tissue sections were stained with Alcian blue and Periodic acid-Schiff (AB/PAS) stains as previously described.^8^ For fluorescent staining, antigen retrieval was performed by immersion of sections in 10 mM citrate buffer (95°C, 30 min). Sections were washed in PBS, permeabilized for 5 min using 0.1% vol/vol Triton X-100 (Merck), and blocked using 5% vol/vol FCS. To detect *C*. *rodentium* ICC169, sections were incubated overnight at 4°C with rabbit anti-O152 primary antibody (1:100, Denka Seiken). Sections were washed in PBS and stained with goat anti-rabbit Alexa 488-conjugated secondary anti-bodies (1:2,000; ThermoFisher) for 2 h at room temperature. Lastly, slides were washed with PBS and counterstained with a mixture of Hoechst DNA dye (5 µg/mL; Merck) and UEA1-DyLight647 conjugated lectin (10 µg/mL, Vectorlabs) for 15 min. Slides were rinsed in dH_2_O, coverslipped using ProLong Gold Antifade mountant (Thermofisher) and imaged using an LSM700 confocal microscope (Zeiss).

#### Citrobacter rodentium distribution analysis

Whole tissue sections stained for *C*. *rodentium* (see previous methods section) were imaged with an LSM700 confocal microscope (Zeiss) using the tile scan function. Raw.czi files were imported into Imaris (v.9.5.0; Bitplane) and converted into.ims format for analysis. Tissue spatial data was manually mapped based on DNA (Hoeschst) signal to identify villus tip and crypt base locations. Distances between the villus tips and nearest crypt base were used to calculate villus length. *C*. *rodentium* was automatically localised using the Imaris spots function to identify O152 positive cells. Distances between individual *C*. *rodentium* cells and nearest crypt base were calculated and cells with a distance to crypt ≤ villus length were categorised as intervillus *C*. *rodentium*. An example of this approach is illustrated in Figure S4G.

#### Fluorescence *in situ* hybridisation

FISH staining for bacterial 16S rRNA was performed using the tissue sections described above. Sections were deparaffinized by sequential washing in Xylene substitute (20 min at 60°C; Merck), 100% ethanol (5 min), and 95% ethanol (5 min). Slides were air dried and flooded with hybridization buffer (40% vol/vol formamide, 0.1% wt/vol SDS, 0.9 M NaCl, and 20 mM Tris, pH 7.4) supplemented with Alexa 555–labelled universal bacterial FISH probe EUB338^40^ (1 mM). Slides were incubated at 37°C overnight in a RapidFISH Slide Hybridization Oven (Boekel Scientific), subsequently submerged in wash buffer (0.9 M NaCl and 25 mM Tris, pH 7.4), and incubated for 20 min at 50°C. Lastly, slides were rinsed in double-distilled water and counterstained with Hoechst dye (5 μg/mL; Merck) and UEA1-FITC conjugated lectin (10 μg/mL, Vectorlabs) for 15 min. Stained slides were imaged using an LSM700 confocal microscope (Zeiss).

#### Quantification of luminal and mucosal bacteria by 16S rRNA gene qPCR

For assessment of bacterial density in different intestinal regions and compartments, approximately 3 cm jejunal, ileal and distal colonic tissues were dissected and flushed with 4 mL 0.22 µm filter-sterilised PBS. Segment content (luminal samples) and flushed tissue (mucosal samples) were collected separately under aseptic conditions and with clean/sterile dissection equipment to prevent sample cross contamination. Luminal samples were immediately stored at −20°C prior to DNA extraction. Mucosal samples were opened longitudinally to expose the mucosal epithelium and transferred to 1 mL filter-sterilized PBS. Mucosal tissue cells were selectively lysed by brief homogenization using an Ultra-Turrax T10 dispersing instrument (IKA) that was sequentially cleaned in RBS detergent (Merck), 70% ethanol and filter-sterilised ddH_2_O between samples. Tissue lysates were centrifuged at 10,000 RCF for 10 min to pellet bacterial cells and tissue debris, and the lysate supernatant was discarded. Bacterial pellets were stored at −20°C prior to further processing. DNA was extracted from both luminal and mucosal samples using a QIAmp PowerFecal Pro kit (Qiagen) with 4x rounds of 4.5 m/s for 40 s bead-beating using a Fast-Prep System (MPBio). DNA extractions were analyzed by qPCR using SsoFast EvaGreen Supermix (Bio-Rad) with 0.3 µm universal primers 926f (5ʹ-AAACTCAAAKGAATTGACGG-3ʹ) and 1062r (5ʹ-CTCACRRCACGAGCTGAC-3ʹ) with 45 ng template DNA. Reactions were performed and monitored using a CFX96 platform (Bio-Rad). Absolute bacterial 16S copy number was quantified using standard curves generated from qPCR of whole 16S gene amplicons purified from *E*. *coli*, and data was normalised to initial sample (luminal content or mucosal tissue) mass.

#### Quantification of mucus bactericidal activity

Soluble mucus protein (SMP) was prepared from freshly dissected small intestinal tissues. Tissues were collected and gently flushed with ice-cold 10 mM sodium phosphate (SP) buffer to remove luminal content. Flushed tissues were opened longitudinally, pinned mucosa side up to a dissection dish and mucus was collected using a micropipette. SMP was prepared by vortexing samples for 10 min and collection of supernatant after centrifugation at 6000 RCF for 10 min. Protease inhibitors (1 mM EDTA, 2x cOmplete Protease Inhibitor Cocktail; Merck) were added to SMP, which was quantified by BCA assay (Pierce) aliquoted and stored at −20°C until use. Bacterial cells used for bactericidal activity testing (*C*. *rodentium* ICC169 and *E*. *coli* K12 W3110) were prepared from overnight cultures that were inoculated into fresh LB medium and grown to OD600 0.5. Bacteria were diluted to an OD600 of 0.2 and washed twice by centrifugation and resuspension in 10 mM SP buffer supplemented with 1 mM EDTA. Sytox Green (2 µM; ThermoFisher) was added to bacterial cell suspensions and incubated in the dark for 10 min, after which 100 μL was distributed into wells of a back 96-well plate. Bacteria were t reated with 100 μL SMP preparations adjusted to 500 μg/mL total protein concentration or 100 μg/mL Polymyxin B (Merck) antibiotic. Sytox Green fluorescence was read at 30 s intervals over 1h in a SpectraMax plate reader (Molecular Devices), and data was normalized to signal at t0.

### QUANTIFICATION AND STATISTICAL ANALYSIS

Statistical details for each experiment are described in the figure legend. For each experiment, ‘‘n’’ refers to the number of biological replicates (animals) used. All histograms present median with interquartile range. Statistical testing of 16S rRNA gene sequencing results was performed in QIIME2^41^ v.2020.11 (Figures 2A, 2B, 5G, and S1B) or using Linear discriminant analysis Effect Size^46^ (Figures 2C and 6B). Statistical testing of MS proteomic data was conducted using Perseus^48^ v1.6.2.2 (Figures 3B and S2B and S2E) or in R v4.1.1 using the package Vegan v2.5–7 (Figures 3A and S2A). All other statistical testing was performed in Prism v.9.4.1 (GraphPad). Non-parametric tests were used in all cases and no method was used to predetermine experimental sample sizes.

## Supplementary Material

1

2

3

4

## Figures and Tables

**Table:** KEY RESOURCES TABLE

REAGENT or RESOURCE	SOURCE	IDENTIFIER
Antibodies		

Rabbit *E*. *coli* O 152 antiserum	Denka Seiken	295774
Goat anti-Rabbit IgG (H + L) Cross-Adsorbed Secondary Antibody, Alexa Fluor^™^ 488	ThermoFisher	A-11008; RRID:AB_2633280

Bacterial and virus strains		

*Citrobacter rodentium* strain ICC169	Sara K Linden	N/A
*Escherichia coli* K12 strain W3110	Fredrik Bäckhed	N/A

Chemicals, peptides, and recombinant proteins		

Autoclavable Mouse Breeder Diet	LabDiet	5021
Fat adjusted diet	Envigo	TD.96132
Ampicillin	Merck	A9518-100G
Metronidazole	Merck	M3761-5G
Vancomycin	Merck	V2002-5G
Neomycin	Merck	N6386-100G
Isoflurane	Kronans apotek	N01AB06
SYTO^™^ 9 Green Fluorescent Nucleic Acid Stain	ThermoFisher	S34854
FluoSpheres^™^ Carboxylate-Modified Microspheres	ThermoFisher	F8816
Lysing matrix E	MPBio	116914100
cOmplete Protease Inhibitor Cocktail	Roche	11873580001
GuHCl 8M	ThermoFisher	24115
DTT	Merck	D9163
Iodoacetamide	Merck	I6125
LysC	Wako	125-05061
Trypsin	Promega	V5111
Ulex Europaeus Agglutinin I (UEA I), DyLight^™^ 649	Vectorlabs	DL-1068-1
Wheat Germ Agglutinin (WGA), Rhodamine	Vectorlabs	RL-1022
MacConkey Agar	ThermoFisher	CM0007B
Luria Broth Base	ThermoFisher	12795027
Nalidixic acid	Merck	N8878-5G
Xylene Substitute	Merck	A5597
Hoechst 34580	Merck	63493
cOmplete^™^ Protease Inhibitor Cocktail	Merck	11697498001
SYTOX^™^ Green Nucleic Acid Stain	ThermoFisher	S7020
Polymyxin B Sulfate	Millipore	5291

Critical commercial assays		

Five Prime Hot Master Mix	Quantabio	733-2474
NucleoSpin Gel and PCR Clean-up kit	Macherey-Nagel	740609
Quant-iT PicoGreen dsDNA kit	ThermoFisher	P11496
Agencourt AMPure XP	Beckman Coulter	A63880
QIAamp PowerFecal Pro DNA Kit	Qiagen	51804
SsoFast^™^ EvaGreen^®^ Supermix	Bio-Rad	1725203
Pierce^™^ BCA Protein Assay Kit	ThermoFisher	23225

Deposited data		

Silva v.138 reference sequence database	Quast et al.^37^	N/A
Mass spectrometry proteomics data	PRIDEpartner repository	PXD028613
16S DNA sequencing data	ENA sequence read archive	PRJEB47610

Experimental models: Organisms/strains		

C57BL/6J	In house	N/A
C57BL/6J	Charles River	000664 |Black 6
Muc2^tm1Avel^	Velcich et al.^17^	N/A
Tgm2^tm1Gml^	Laurenzi & Melino^38^	N/A

Oligonucleotides		

V4 region 515F and 806R primers	Kozich et al.^39^	N/A
EUB338 16S FISH probe (Alexa Fluor^™^ 555 conjugated)	Amann et al.^40^	N/A

Software and algorithms		

Zen (version 2.3)	Carl Zeiss	http://www.zeiss.com
Imaris ×64 (version 9.5.0)	Oxford Instruments	http://imaris.oxinst.com/
Prism (version 9.4.1)	GraphPad	http://www.graphpad.com/
QIIME 2 (version 2020.11)	Bolyen et al.^41^	N/A
DADA2	Callahan et al.^42^	N/A
MAFFT (version 7.407)	Katoh et al.^43^	N/A
FastTree 2	Price et al.^44^	N/A
q2-feature-classifier	Bokulich et al.^45^	N/A
LEfSe	Segata et al.^46^	N/A
MaxQuant (v1.5.7.4)	Cox & Mann^47^	N/A
Perseus (v1.6.2.2)	Tyanova et al.^48^	N/A
StavroX (version 3.6.6)	Gotze et al.^49^	N/A
R (v4.1.1)	The R Project for Statistical Computing	https://www.r-project.org/

Other		

Laser scanning confocal imaging system	Carl Zeiss	LSM 700
Illumina MiSeq	Illumina	http://www.illumina.com/systems/sequencing-platforms/miseq.html
Nanosep 10K Omega	Pall Life Sciences	OD010C35
EASY-nLC system 1000	ThermoFisher	LC120
reverse-phase column (150 × 0.075 µm inner diameter, C18-AQ 3 µm	In-house	N/A
QExactive Hybrid Quadrupole-Orbitrap Mass Spectrometer	ThermoFisher	IQLAAEGAAPFALGMAZR
T 10 basic ULTRA-TURRAX^®^	IKA	0003737000
RapidFISH Slide Hybridizer	Boekel Scientific	240200
FastPrep-24^™^	MP Biomedicals	116004500
CFX96 Touch Real-Time PCR Detection System	Bio-Rad	1845097
SpectraMax^®^ M2 Multimode microplate reader	Molecular Devices	https://www.moleculardevices.com/products/microplate-readers/multi-mode-readers/spectramax-m-series-readers

## Highlights

- Western-style diet disrupts the mucus layer in the small intestinal jejunum
- The mucus layer is an essential habitat for the jejunal microbiota
- Jejunal microbiota provides resistance against enteric pathogen colonization
- Mucus supports colonization resistance by providing a niche for the microbiota
