# Supplementary material for: Muc2-dependent microbial colonization of the jejunal mucus layer is diet sensitive and confers local resistance to enteric pathogen infection
Source: Cell Rep. Author manuscript; Available in PMC 2023 Oct 23. (PMC10404306; doi:10.1016/j.celrep.2023.112084)
Supplement: 1 [file NIHMS1878880-supplement-1.pdf]

Cell Reports, Volume *42*

Supplemental information

Muc2-dependent microbial colonization of the jejunal mucus layer is diet sensitive and confers local resistance to enteric pathogen infection

George M.H. Birchenough, Bjoern O. Schroeder, Sinan Sharba, Liisa Arike, Christian V. Recktenwald, Fabiola Puértolas-Balint, Mahadevan V. Subramani, Karl T. Hansson, Bahtiyar Yilmaz, Sara K. Lindén, Fredrik Bäckhed, and Gunnar C. Hansson

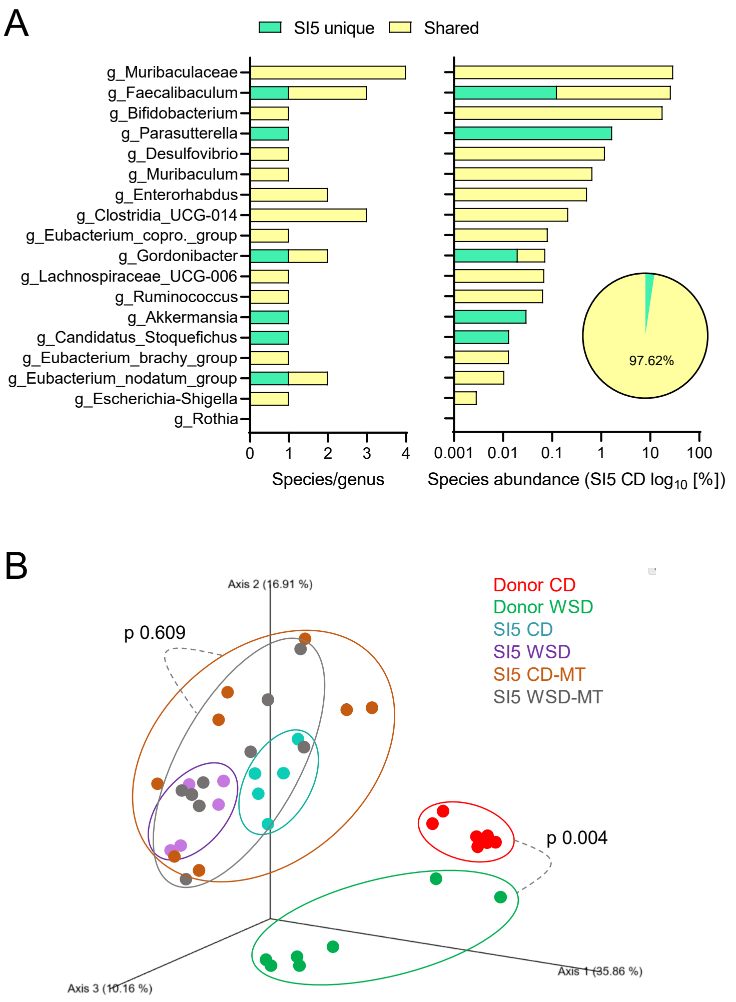

Supplemental Figure 1: Microbiota from CD-fed donors fails to engraft in SI5 of WSD-fed mice (related to Figure 2)

A: Species level ASVs belonging to bacterial genus level taxons detected in 16S rRNA gene sequencing of CD-fed SI5 and caecal donor samples shown in Fig. 2F. Graphs show the number of SI5 unique and SI5/caecum shared species per genus (left) and relative abundance of unique and shared species in SI5 (right). Inset pie chart shows the overall proportion of SI5 unique and SI5/caecum shared 16S reads detected.

B: Principal component analysis of β-diversity (Bray Curtis) of bacterial communities in CD or WSD-fed donor (caecal contents) or SI5 samples after microbiota transfer. Significance by Pairwise PERMANOVA.

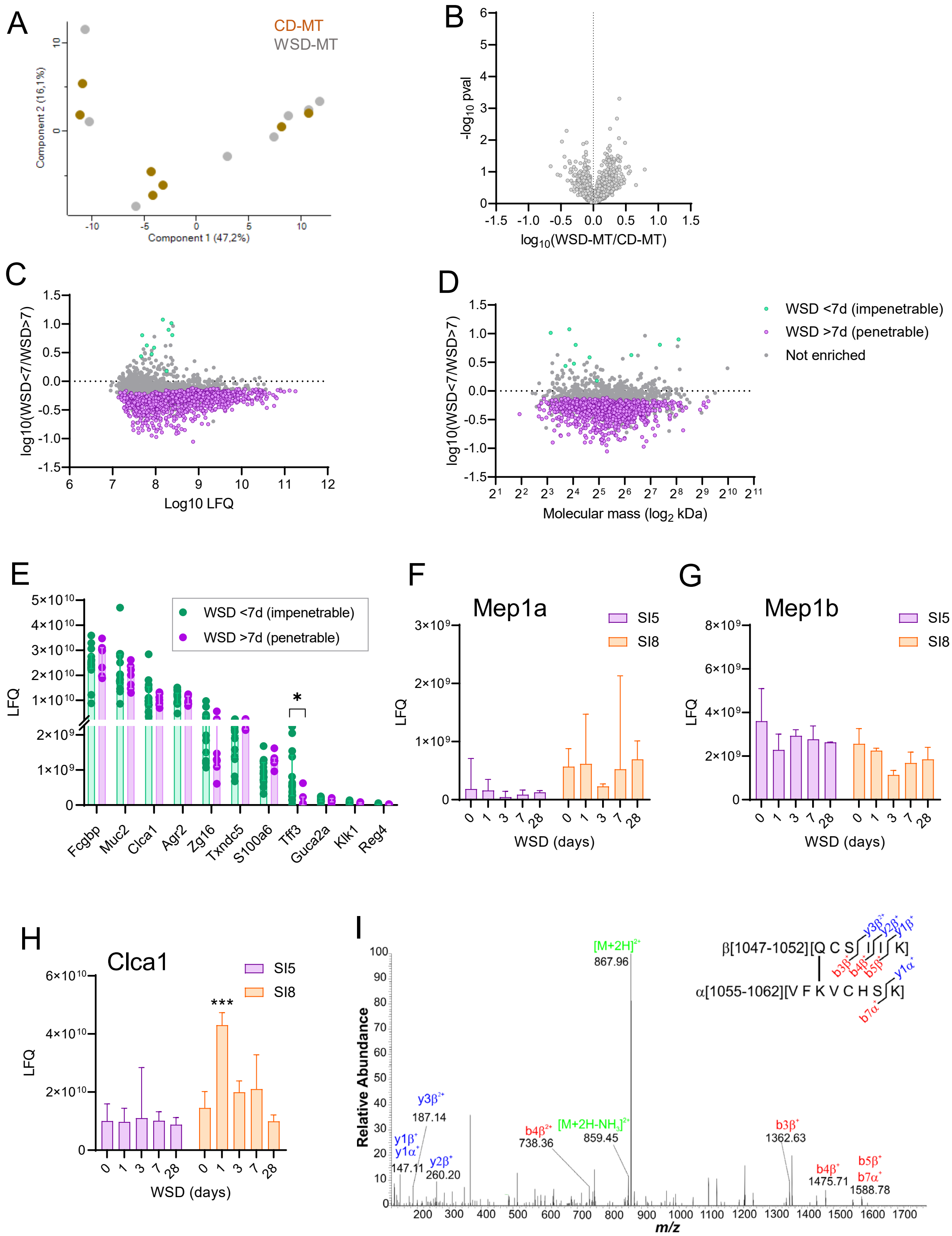
A
CD-MT
WSD-MT
Component 2 (16,1%)
Component 1 (47,2%)
B
-log10 pval
log10(WSD-MT/CD-MT)
C
log10(WSD<7/WSD>7)
Log10 LFQ
D
WSD <7d (impenetrable)
WSD >7d (penetrable)
Not enriched
Molecular mass (log2 kDa)
E
WSD <7d (impenetrable)
WSD >7d (penetrable)
LFQ
Fcgbp
Muc2
Clca1
Agr2
Zg16
Txndc5
S100a6
Tff3
Guca2a
Klk1
Reg4
F
Mep1a
SI5
SI8
WSD (days)
G
Mep1b
H
Clca1
I
Relative Abundance
m/z
β[1047-1052][Q C S I I K]
α[1055-1062][V F K V C H S K]
[M+2H]2+
867.96
[M+2H-NH3]2+
859.45
y3β2+
187.14
b4β2+
738.36
b3β+
1362.63
b4β+
1475.71
b5β+
b7α+
1588.78
y1β+
y1α+
147.11
y2β+
260.20

Supplemental Figure 2: Proteomic analysis of small intestinal mucus from CD and WSD-fed mice (related to Figure 3)

A-B: Mass spectrometry based label-free quantification (LFQ) of SI5 mucus proteomes from CD-MT and WSD-MT mice. Principal component analysis (A) and volcano plots (B) comparing protein abundance ratios.

C-D: Smear plots showing the distribution of proteins enriched in SI5 from WSD <7d compared to WSD >7d fed mice in relation abundance (C; LFQ intensity) or protein molecular weight (D); related to Fig. 3B.

E-H: Mass spectrometry based label-free quantification (LFQ) of core mucus proteins (E) from SI5 mucus of mice from different phenotype groups, or indicated proteins (Mep1a, Mep1b, Clca1) from SI5 and SI8 mucus of WSD fed mice. Data shows median and interquartile range of n=2-8 replicates. Significance by Welch's t-test and Permutation-based FDR (E) or Dunnett's test (F-H) (* $p<0.05$, ** $p<0.01$). (* $p<0.05$, ** $p<0.01$, *** $p<0.0001$).

I: Annotated MS2 fragment spectrum of the parent ion $[M+2H]^{2+}$ 867.955 showing the isopeptide-bond cross-link between Gln1047 and Lys 1057. B ions are labelled in red and y ions are labelled in blue. The non-fragmented precursor ions are labelled in green.

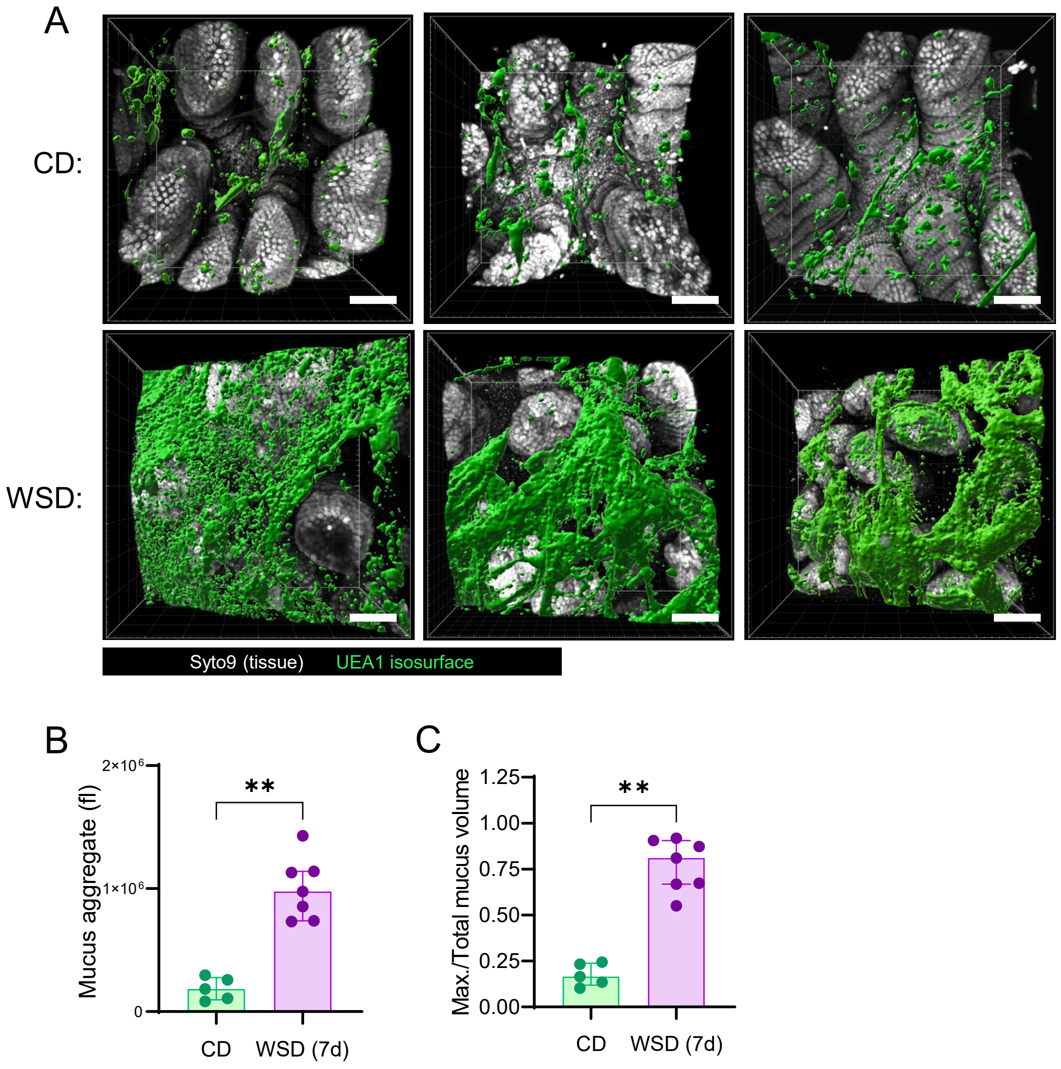

Supplemental Figure 3: Volumetric analysis of SI5 mucus aggregation (related to Figure 3)

A: Isosurface mapping of UEA1 fluorescence in three representative SI5 tissues from CD and WSD (7d) fed mice based on ex vivo mucus imaging experiments. Scale bars are 50 μm.

B-C: Quantification of mucus aggregation in SI5 tissue from CD and WSD (7d) fed mice based on ex vivo mucus imaging experiments shown in (A) by isosurface mapping of lectin fluorescence. Absolute volume of aggregated mucus per z-stack image (B). Relative contribution of largest mucus aggregate to total aggregation volume (C). Data shows median and interquartile range for n=5-7 mice per group; significance by Mann-Whitney (** $p<0.01$).

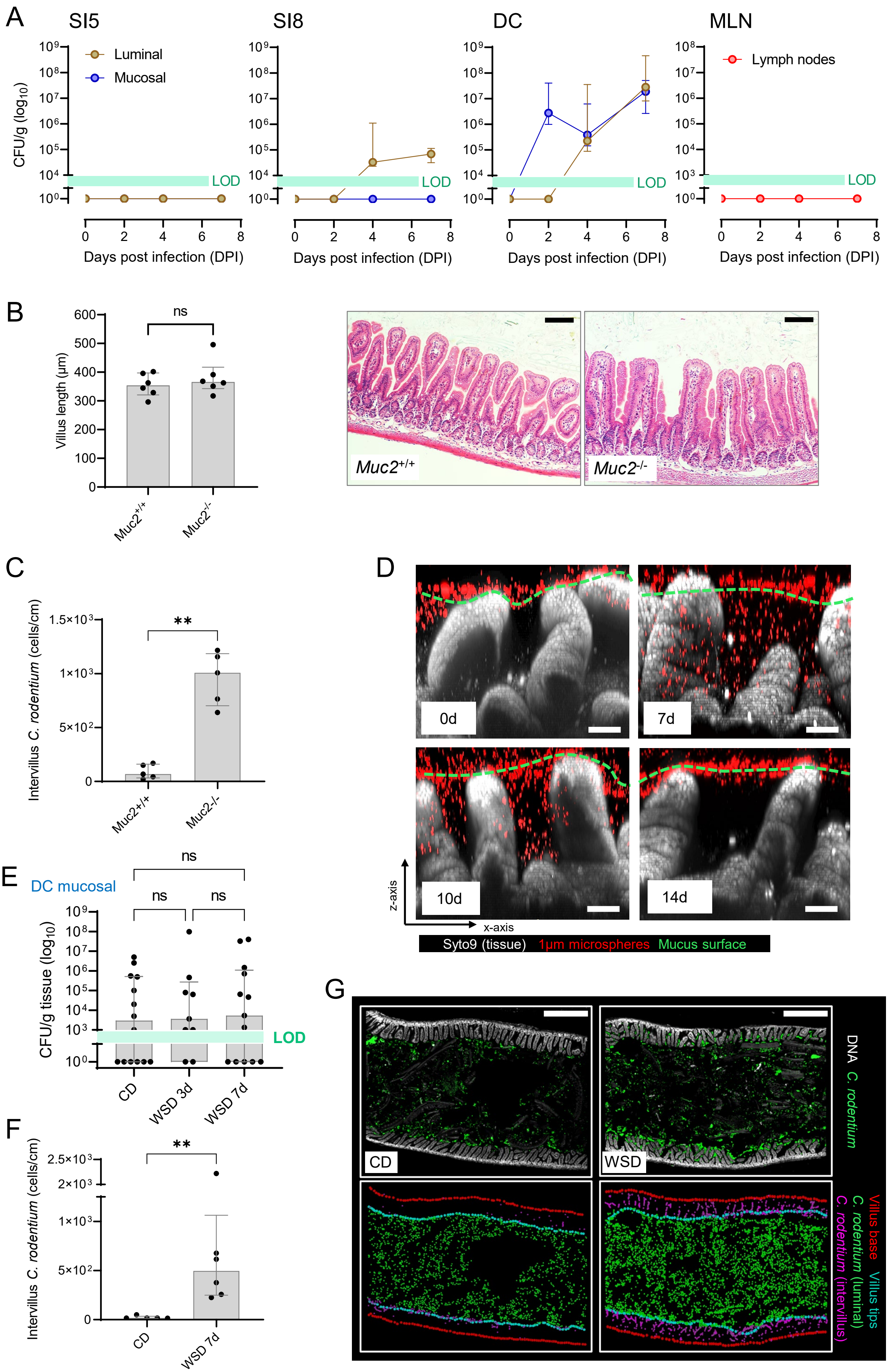

A
SI5
SI8
DC
MLN
Luminal
Mucosal
Lymph nodes
CFU/g (log10)
LOD
Days post infection (DPI)
B
ns
Villus length (µm)
Muc2+/+
Muc2-/-
C
**
Intervillus C. rodentium (cells/cm)
Muc2+/+
Muc2-/-
D
0d
7d
10d
14d
z-axis
x-axis
Syto9 (tissue) 1µm microspheres Mucus surface
E
DC mucosal
ns
ns
ns
CFU/g tissue (log10)
LOD
CD
WSD 3d
WSD 7d
F
**
Intervillus C. rodentium (cells/cm)
CD
WSD 7d
G
CD
WSD
DNA C. rodentium
Villus base Villus tips
C. rodentium (luminal)
C. rodentium (intervillus)

Supplemental Figure 4: Colonization of SI5 by *Citrobacter rodentium* (related to Figure 4)

A: Colonization kinetics of *C. rodentium* in different intestinal regions (SI5, SI8 and DC) and mesenteric lymph nodes (MLN) of WT C57BL/6 mice. Data shows median and interquartile range of CFU/g luminal content or mucosal tissue from n=4-5 mice per time point. LOD: limit of detection.

B: Comparison of *Muc2*$^{+/+}$ and *Muc2*$^{-/-}$ SI5 tissues showing H&E stained tissue sections and quantification of villus length. Scale bars are 100 µm. Data shows median and interquartile range from n=6 replicates per group, significance by Mann-Whitney (ns: not significant).

C: Analysis of *C rodentium* distribution in infected *Muc2*$^{+/+}$ and *Muc2*$^{-/-}$ SI5 tissue sections. Data shows median and interquartile range from n= replicates per group, significance by Mann-Whitney (** p<0.01). Related to Fig. 4F.

D: Confocal z-stacks showing x/z-axis cross sections of SI5 tissue (grey) and 1µm microspheres (red) from mice before and after WSD exposure; approximate mucus surface (green dashed line) indicated. Scale bars are 50 µm. Related to Fig. 4H.

E: Infection of CD, WSD 3d and WSD 7d fed WT mice with *C. rodentium*. CFU enumeration from DC mucosal samples. Significance by Dunn's multiple comparison (ns: not significant). Data shows median and interquartile range from two independent experiments, n=4-7 mice per group per experiment. LOD: limit of detection.

F-G: Analysis of *C rodentium* distribution in infected CD-fed and WSD-fed SI5 tissue sections. Quantification of intervillus *C. rodentium* (F) by image analysis is illustrated (G). Panels in (G) show raw fluorescence data (upper) and processed analysis (lower) examples, scale bars 500 µm. Data shows median and interquartile range from n=5-6 replicates per group, significance by Mann-Whitney (** p<0.01). Related to Fig. 4M.

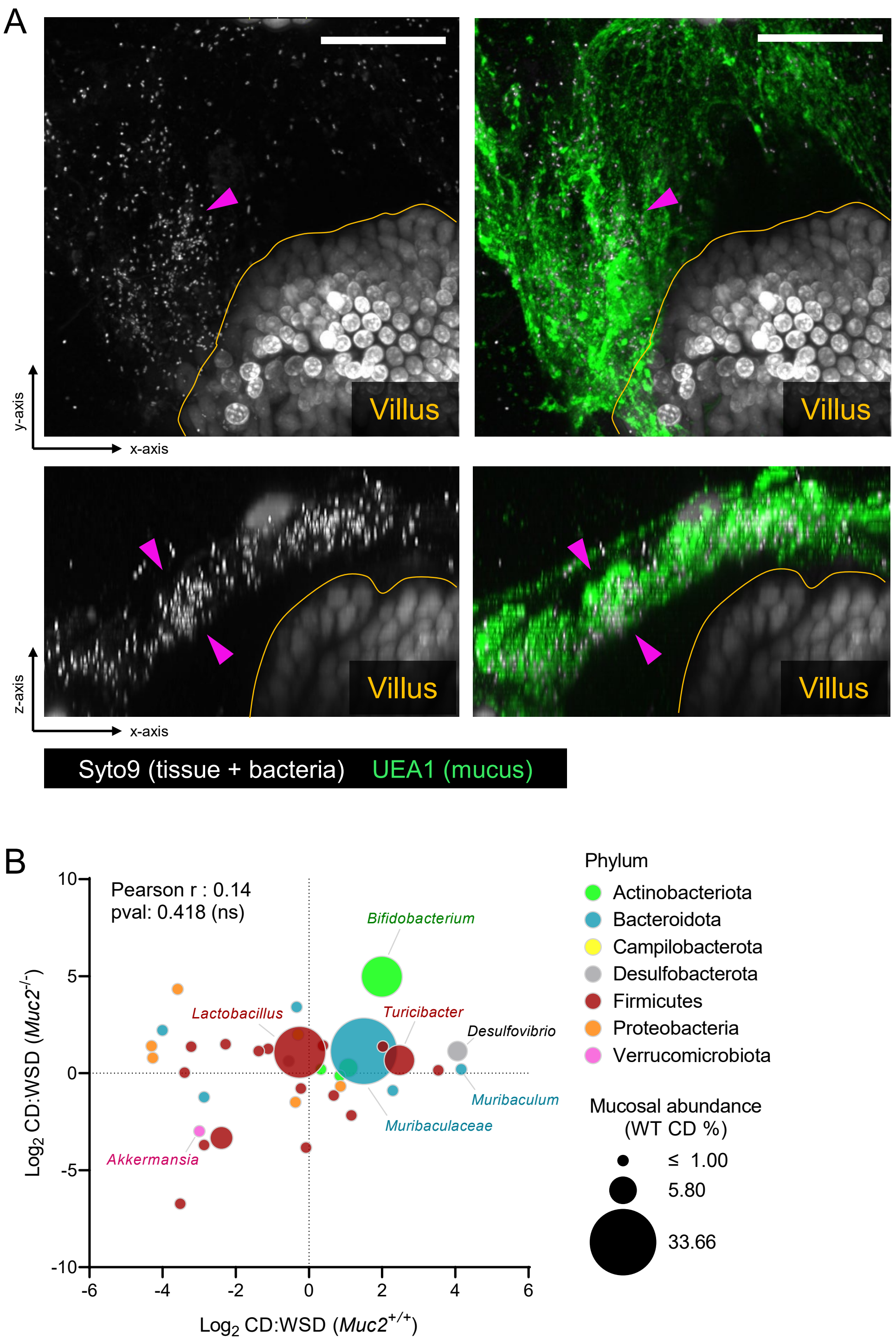

Supplemental Figure 5: Microbiota-mucus interactions after WSD-expsoure (related to Figure 5)

A: *Ex vivo* confocal microscopy imaging of SI5 tissue and bacteria (grey) and mucus (UEA1; green) in WSD fed mice. Images are confocal z-stacks showing x/y-axis (upper panels) and x/z-axis cross sections. Villus epithelial surface (yellow) and mucus embedded bacterial cells (magenta arrows) are indicated. Scale bars are 30 µm.

B: Comparison of $\log_2$ CD:WSD (Muc2$^{+/+}$) and $\log_2$ CD:WSD (Muc2$^{-/-}$) abundance ratios of bacterial taxa detected in all groups (H); data points represent individual taxa colour coded by phylum and sized by median mucosal relative abundance (RA) in CD fed Muc2$^{+/+}$ mice.

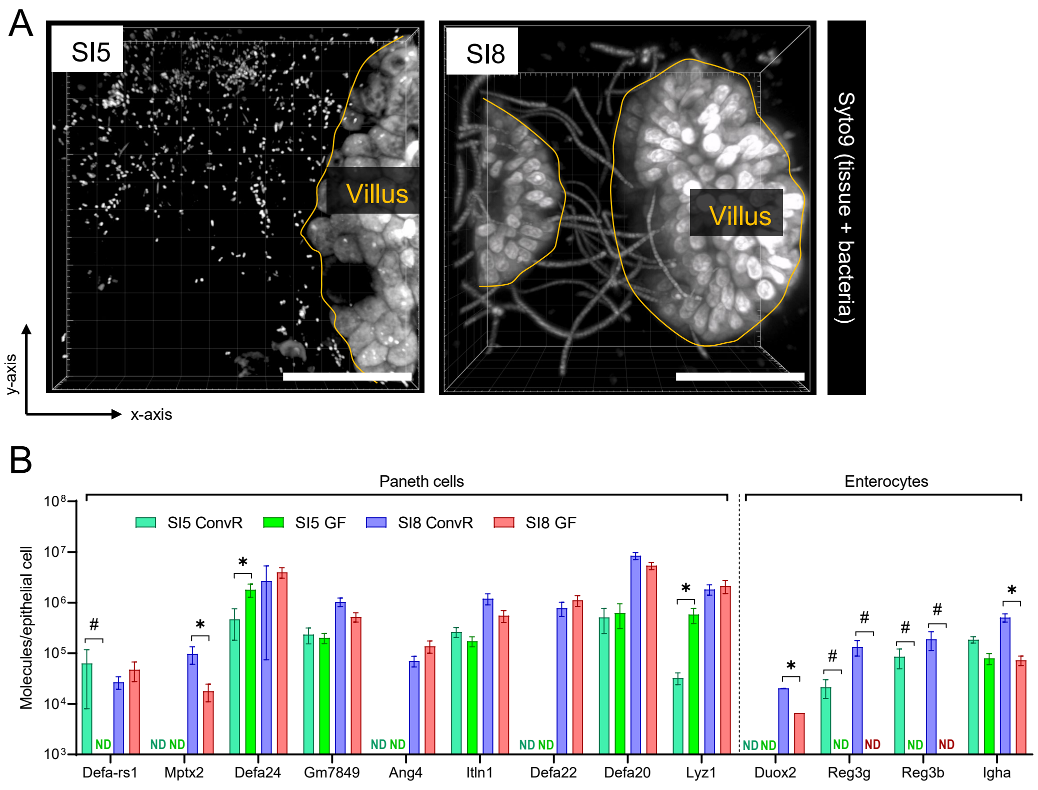

Supplemental Figure 6: Microbiota and host factors differentiating the SI5 and SI8 mucosal niche (related to Figure 6)

A: Unprocessed images used to generate Fig. 6A. Images are confocal z-stacks showing x/y-axis cross sections of Syto9 cell dye (grey) staining in SI5 and SI8 tissue from CD fed mice. Villus epithelial surface (yellow) is indicated. Scale bars are 40 µm.

B: SI5 and SI8 epithelial proteomics data from ConvR and GF mice extracted from Arike *et al.* 2020. Data shows mean and standard deviation of estimated protein molecules per cell for n=4 mice per group; ConvR vs. GF comparisons where $p < 0.05$ (*) or where proteins are detected in ConvR but not GF (#) are indicated. Related to Fig. 6E.

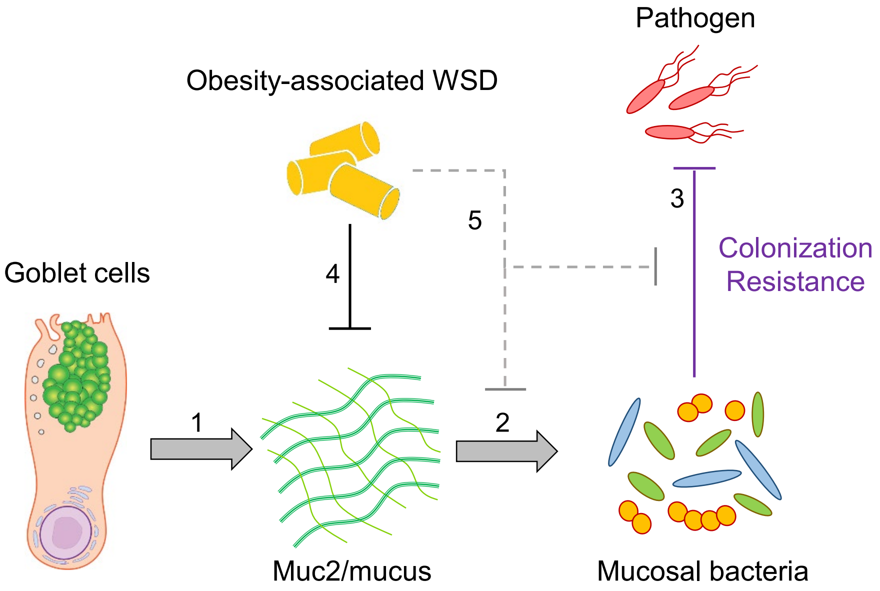

Supplemental Figure 7: Simplified schematic illustrating how the host supports jejunal colonization resistance and how this is affected by diet (related to Discussion)

(1) Jejunal goblet cells secrete Muc2 polymers, which form the jejunal mucus layer. (2) Jejunal mucus supports colonization by a consortium of mucosal bacteria. (3) Bacterial colonization of the jejunal mucus provides direct colonization resistance against incoming pathogens. (4) An obesity-associated Western-style diet prevents normal formation of the jejunal mucus layer. (5) Inhibition of mucus layer formation indirectly suppresses jejunal mucosal bacteria resulting in loss of jejunal colonization resistance to pathogens.
